# Burden of heart failure in Asian Countries from 1990 to 2021: Update from the Global Burden of Disease Study 2021

**DOI:** 10.1371/journal.pone.0352930

**Published:** 2026-07-29

**Authors:** Chaofeng Niu, Donghua Xue, Juwei Dong, Qiwen Yang, Peiyu Zhang, Lan Wei, Nina Wang, Meng Li, Lijing Zhang

**Affiliations:** Department of Cardiology, Dongzhimen Hospital, Beijing University of Chinese Medicine, Beijing, China; Kerman University of Medical Sciences, IRAN, ISLAMIC REPUBLIC OF

## Abstract

Heart failure (HF) remains a major public health challenge in Asia, with rising prevalence and disability burden. This study analyzed data from the 2021 Global Burden of Disease study (GBD 2021) to assess HF trends across 48 Asian countries from 1990 to 2021. In 2021, there were 29.5 million HF cases in Asia, a 155% increase since 1990, with an age-standardized prevalence rate rising from 583.62 to 633.76 per 100,000. Years lived with disability (YLDs) also surged by 155%, reaching 2.86 million in 2021. China accounted for 44.34% of Asia’s HF cases, with the highest YLDs. Treated HF cases were most common, but severe HF contributed most to YLDs. The middle-high socio-demographic index (SDI) region had the highest age-standardized prevalence rate, while most SDI regions saw increasing trends, except Japan and Cyprus. The findings highlight the growing HF burden in Asia, urging targeted interventions to address this escalating health crisis.

## 1. Introduction

Heart failure (HF) is a complex clinical syndrome with different underlying etiologies consisting of cardinal symptoms accompanied by multiple signs and comorbidities [1].As a major public health issue, HF affected an estimated 56 million people worldwide in 2019 [2].Owing to the rapidly growing ageing population, the prevalence of HF tends to increase [3], greatly increasing the high rate of rehospitalization and economic burden for individuals, families and public healthcare systems globally [4,5].

About 60 percent of the global population, approximately 4.3 billion people, live in Asia, including the world’s most populous countries, China and India [6], highlighting the profound impact of demographic trends on global disease. The HF epidemic is especially pertinent in Asia, contributing to 60% (a total of 31.89 million cases) of the HF cases reported worldwide in 2019 [7]. In China, 1.3% of adults aged ≥35 years, over 4.5 million people, are living with HF [8], while in India, the prevalence of HF is estimated to be between 1.3 and 4.6 million [9]. Furthermore, Asian patients with HF tend to be approximately a decade younger than Western populations, suggesting a higher burden of HF in Asian countries [10].

However, high-quality population-based studies that elucidate the epidemiological landscape of HF in Asia remain limited, with a substantial knowledge gap in prior studies [11]. While current reports primarily focus on individual countries in Asia, comprehensive data on the temporal epidemiological trends of HF across the region are urgently needed.

This study aims to analyze the trends in prevalence and years lived with disability (YLDs) of HF in Asia using data from the 2021 Global Burden of Disease (GBD 2021). The results will help identify disparities in the burden of HF across Asia and provide valuable insights to support healthcare systems in addressing this growing challenge.

## 2. Materials and methods

### 2.1 Data source

The GBD 2021 collaborators conducted a systematic cause-of-death analysis, estimating mortality and YLDs for 288 causes of death by age, sex, location, and year across 204 countries and territories and 811 subnational locations from 1990 to 2021 [12]. This study constituted an independent secondary analysis utilizing data outputs from the GBD 2021 study to specifically report on the burden of HF in the Asia region. We obtained the number of prevalence cases and YLDs, all-age rate and age-standardized rates for HF, stratified by sex (female, male, and both), age (5–95 + years old), year (from 1990 to 2021), and Socio-demographic index (SDI) in Asia region from the GBD 2021. The data used in this study were downloaded from the GBD Results Tool of the Global Health Data Exchange (http://ghdx.healthdata.org/gbd-results-tool) on December 20, 2024. None of the data contain information that could identify individual subjects.

This study constitutes a secondary analysis of publicly available, fully anonymized, and aggregated data. According to Article 32, Items 1 and 2 of the “Administrative Measures for Ethical Review of Life Science and Medical Research Involving Humans” (issued on February 18, 2023), research that (1) uses legally obtained public data and (2) uses anonymized information is exempt from ethical review. Therefore, neither institutional review board approval nor informed consent was required. The datasets and analysis code for this study are available in a figshare repository (https://doi.org/10.6084/m9.figshare.30451526).

### 2.2 Definition of HF

The definition of HF was updated in the GBD 2021 study from that used in GBD 2019 [13]. HF was diagnosed clinically using the Framingham or European Society of Cardiology criteria, which can be found in a previous study [14]. In this study, we used the GBD 2021 definition of HF, which is stage C or higher as defined by the Universal Definition of HF, to capture individuals who are currently symptomatic and those who have been diagnosed with HF but are currently asymptomatic. Briefly, Framingham criteria require patients with HF to fulfill 2 major criteria or 1 major criterion and 2 minor criteria. According to European Society of Cardiology criteria, typical signs and symptoms caused by structural and/or functional cardiac abnormality are necessary [15].

### 2.3 HF severity categories and disability weights

The GBD 2021 study provides estimates for HF disaggregated into four mutually exclusive severity categories: Treated, Mild, Moderate, and Severe. These categories are defined based on clinical symptoms and are operationalized to correspond with New York Heart Association (NYHA) functional classifications for population-level modeling. The prevalence estimates for each severity category were obtained directly from the GBD 2021 Results Tool by selecting the impairment “Heart Failure” and applying the “Severity” split. The specific disability weights (DWs) for each category, as defined in the GBD 2021 disability weights list, are as follows: (1) Treated HF: This category represents cases controlled with medication. A DW of 0.049 (95% UI: 0.031–0.072) was applied, representing minimal health loss due to successful treatment. (2) Mild HF: This category corresponds to NYHA class I. A DW of 0.042 (95% UI: 0.026–0.062) was applied. (3) Moderate HF: This category corresponds to NYHA class II. A DW of 0.072 (95% UI: 0.047–0.103) was applied. (4) Severe HF: This category corresponds to NYHA classes III and IV. A DW of 0.179 (95% UI: 0.122–0.251) was applied.

The YLDs for each severity category were calculated within the GBD modeling framework were calculated by multiplying the prevalence estimate for that category by its corresponding DW. The total YLDs for HF as a cause are the sum of the YLDs from these four severity categories: Total HF YLDs = YLDs-Treated + YLDs-Mild + YLDs-Moderate + YLDs-Severe.

In this study, we extracted the number of prevalence cases, prevalence rates, number of YLDs, and YLD rates for each of these four severity categories directly from the GBD 2021 Results Tool to analyze their respective burdens and trends.

### 2.4 Asian regions

The regional groupings and sub-regional divisions in this study strictly adhere to the United Nations geoscheme for Asia. No sub-regions were excluded or combined. We analyzed the GBD burden across five Asian regions: Central Asia, East Asia, Southeast Asia, South Asia and Western Asia. A total of 48 countries and territories were included, as follows: Central Asia comprises Kazakhstan, Turkmenistan, Uzbekistan, Kyrgyzstan, and Tajikistan; East Asia encompasses Japan, Republic of Korea, China, Taiwan (Province of China), Democratic People’s Republic of Korea, and Mongolia; Southeast Asia includes Brunei Darussalam, Singapore, Malaysia, Indonesia, Philippines, Thailand, Viet Nam, Lao People’s Democratic Republic, Cambodia, Myanmar, and Timor-Leste; South Asia has Sri Lanka, Iran (Islamic Republic of), Bhutan, Bangladesh, India, Maldives, Afghanistan, Nepal, and Pakistan; and Western Asia includes Cyprus, Kuwait, Qatar, United Arab Emirates, Bahrain, Georgia, Israel, Jordan, Lebanon, Oman, Saudi Arabia, Armenia, Azerbaijan, Iraq, Syrian Arab Republic, Palestine, and Yemen.

### 2.5 Definition of HF attribution

The etiology of HF is complex and typically involves a range of cardiovascular and systemic conditions. In the GBD 2021 study, the etiologies of HF include ischemic heart disease, hypertensive heart disease, chronic obstructive pulmonary disease, pulmonary hypertension, chronic kidney disease, and various systemic diseases. The selection criteria and detailed definitions for each etiology are described in previously published studies [12,16].

### 2.6 Estimated annual percentage change

The Estimated Annual Percentage Change (EAPC) is a commonly utilized approach for quantifying trends during a defined time frame [17]. This study utilized two distinct approaches to calculate the EAPC for Asian countries and regions [18]. (1) EAPC of age-standardized rates (EAPC-ASR): This metric describes the average annual percentage change in the age-standardized rate (ASR), which reflects trends independent of changes in population size and age structure. It is the preferred measure for assessing changes in disease risk at a population level. (2) EAPC of All-Age Counts (EAPC-Count): This metric describes the average annual percentage change in the all-age number of cases or YLDs. It captures the compound effect of changes in disease risk, population growth, and population aging, representing the absolute growth in the burden faced by health systems.

Both EAPCs were calculated by fitting a linear regression model to the natural logarithm of the respective outcome variable (either ASR or Count). The generic model specification was:


$$\begin{array}{c} \text{ln}(\text{Y})=\alpha +\beta \times \text{t}+\epsilon \end{array}$$

Where Y represents the outcome variable (ASR or Count) for a given year, α is the intercept, t is the time variable (coded as 1990 = 0, 1991 = 1,..., 2021 = 31), β is the regression coefficient, and ε is the error term. The corresponding EAPC was then derived as:


$$\begin{array}{c} \text{EAPC}=\text{100}\times (\text{exp}(\beta )-\text{1}) \end{array}$$

A positive EAPC value indicates an increasing trend, while a negative value indicates a decreasing trend. For each EAPC estimate, we report its 95% confidence intervals (CI) derived from the linear model. Trends were considered statistically significant if the 95% CI did not cross zero. The specific outcome variable used (ASR or Count) is explicitly stated for every EAPC result reported in the study.

### 2.7 Socio-demographic index

Socio-demographic Index (SDI) is an indicator of social and economic condition of a country’s or region’s level of development based on data on fertility rate, education level, and per capita income [7]. SDI ranges from 0 to 1; higher levels indicate greater socioeconomic development. In this study, regions across Asia were classified into 5 SDI regions (low, low-middle, middle, middle-high, and high) to explore the association between the burden of HF and levels of socioeconomic development.

### 2.8 Statistical analysis

Numbers of prevalence, YLDs, and their corresponding age-standardized rates were the main indicators used to describe the burden of HF. Each rate is reported per 100,000 population, along with 95% uncertainty intervals (UI) according to the GBD algorithm [19]. The dynamics of HF were analyzed by calculating EAPCs to identify temporal trends in the disease burden, 95% CI of EAPCs were determined by linear modeling [20,21]. A trend was considered statistically significant if the 95% CI of the EAPC did not cross zero. Gaussian regression models were used to analyze associations among the EAPC, rates, and the SDI of HF in Asia [22]. All calculations were performed using R (version 4.4.2). The code was developed and executed using the RStudio integrated development environment (version 2024.09.1+394).

## 3. Results

### 3.1 Trends in prevalence of HF in Asia and by country

Over the past 32 years, the number of HF cases in the region increased from 11.60 million (95% UI: 10.24 million-13.21 million) to 29.55 million (95% UI: 26.00 million-34.13 million), marking a 155.06% (95% UI: 145.93–164.43%) increase. In 2021, the age-standardized prevalence rate of HF in Asia was 633.76 per 100,000 population (95% UI: 560.53–729.43), reflecting an 8.59% (95% UI: 5.72–11.41%) increase compared to 1990, with an EAPC-ASR of 0.26 (95% CI: 0.24–0.29). A detailed summary of HF prevalence across different regions in Asia is presented in Table 1.

**Table 1:** Prevalence numbers, prevalence rates, YLDs numbers, and YLD rates in 1990 and 2021, along with the Percentage Change and EAPCs of HF from 1990 to 2021 for Asia and its 48 countries. (Red represents heavy burden, and blue represents light burden.

	1990				2021				1990-2021							
	Prevalence		YLDs		Prevalence		YLDs		Prevalence				YLDs			
									Percentage Change		EAPCs		Percentage Change		EAPCs	
	Number (95% UI)	Rate per 100,000, N (95% UI)	Number (95% UI)	Rate per 100,000, N (95% UI)	Number (95% UI)	Rate per 100,000, N (95% UI)	Number (95% UI)	Rate per 100,000, N (95% UI)	Number (95% UI)	Rate (95% UI)	Number (95% CI)	Rate (95% CI)	Number (95% UI)	Rate (95% UI)	Number (95% CI)	Rate (95% CI)
**Global**	25433219.59(22319174.53-29214319.64)	641.141(556.288-745.461)	2429266.59(1644895.72-3347610.91)	61.071(41.341-85.006)	55496832.84(48996497.57-63842413.20)	676.683(598.683-776.837)	5311083.92(3626093.75-7360662.80)	64.700(44.204-89.469)	154.69(145.95-163.75)	5.54(2.70-8.49)	1.28(1.25-1.32)	0.19(0.18-0.20)	118.63(112.58-124.50)	5.94(2.93-8.99)	1.28(1.25-1.32)	0.20(0.18-0.21)
**Asia**	11600993.48(10240997.72-13210701.50)	583.62(508.11-676.59)	1119605.63(762603.36-1528579.79)	56.11(38.46-77.56)	29546578.66(26007058.07-34132066.71)	633.76(560.53-729.43)	2855711.00(1943867.82-3968544.11)	61.08(41.60-84.91)	155.06(145.93-164.43)	8.59(5.72-11.41)	3.08(3.04-3.13)	0.26(0.24-0.29)	155.06(145.93-164.43)	8.86(5.72-11.84)	3.08(3.05-3.12)	0.26(0.24-0.29)
**High SDI**																
Brunei Darussalam	666092.87(584110.25-768886.36)	**439.60(374.37-519.20)**	**64.70(43.57-90.06)**	**41.41(27.78-57.33)**	1404840.88(1246525.51-1601283.37)	446.83(384.65-525.32)	136.09(89.82-185.73)	42.06(28.52-58.05)	109.78(93.85-130.58)	1.64(−4.45-7.95)	2.30(2.26-2.35)	0.04(0.03-0.05)	110.33(90.52-133.55)	1.58(−5.38-9.25)	2.32(2.27-2.36)	0.04(0.03-0.04)
Cyprus	3218.85(2664.75-3897.10)	439.42(371.00-525.02)	304.85(193.86-431.26)	41.48(27.10-58.96)	8410.90(6890.71-10315.20)	465.59(394.39-554.11)	793.88(498.44-1136.71)	43.91(28.59-61.18)	161.30(136.72-186.15)	5.96(−2.23-14.04)	2.62(2.41-2.82)	−0.28(−0.46--0.10)	160.42(133.77-186.44)	5.86(−3.54-14.92)	2.61(2.40-2.82)	−0.27(−0.45--0.09)
Japan	7377.85(6409.37-8416.05)	472.61(419.41-535.48)	64571.64(43657.20-89456.16)	45.56(31.22-62.76)	27741.15(23425.05-32270.72)	459.77(422.32-503.18)	135401.92(92600.59-188306.83)	44.20(30.13-60.91)	110.91(95.82-129.74)	−2.72(−7.52-2.55)	2.39(2.33-2.44)	−0.13(−0.15--0.10)	109.69(93.51-128.94)	−3.00(−8.16-3.13)	2.36(2.30-2.43)	−0.14(−0.17--0.11)
Kuwait	1663.92(1437.43-1917.88)	862.52(721.11-1036.07)	692.24(460.81-962.97)	79.90(52.30-112.22)	12773.17(10821.23-15134.94)	896.16(753.32-1079.93)	2594.10(1717.47-3623.59)	83.49(54.67-114.95)	276.01(247.74-307.36)	3.90(−2.77-12.09)	4.68(4.50-4.87)	0.13(0.10-0.16)	274.74(241.38-312.49)	4.49(−2.93-13.65)	4.69(4.50-4.88)	0.16(0.12-0.20)
Qatar	151903.47(130700.54-175362.49)	801.89(673.05-959.72)	157.44(105.40-218.46)	75.00(49.15-104.12)	433737.72(372554.91-505417.41)	864.94(734.12-1017.07)	1215.17(816.52-1751.70)	81.69(53.93-113.65)	667.65(612.15-733.52)	7.86(0.24-15.63)	7.85(7.34-8.36)	0.29(0.27-0.31)	671.83(600.51-756.50)	8.92(0.88-17.71)	7.89(7.37-8.41)	0.33(0.31-0.36)
Republic of Korea	685.75(598.27-782.44)	512.85(439.07-601.37)	15360.53(10422.16-21058.12)	51.79(35.26-72.02)	1438.55(1236.97-1684.62)	576.44(507.02-657.16)	42961.03(29226.98-58567.18)	56.59(38.21-77.32)	185.54(158.00-215.28)	12.40(3.67-21.15)	3.61(3.50-3.72)	0.44(0.31-0.57)	179.68(150.72-211.85)	9.26(−0.69-19.07)	3.52(3.42-3.63)	0.33(0.20-0.46)
Singapore	6774.14(5905.89-7728.29)	508.09(433.41-589.72)	1038.18(688.56-1422.34)	47.48(31.54-66.55)	45606.15(37435.80-54719.21)	529.30(460.36-616.31)	3538.52(2378.07-4927.21)	49.66(33.36-68.87)	240.32(211.98-269.48)	4.18(−2.23-10.24)	3.96(3.86-4.07)	0.14(0.13-0.14)	240.84(209.76-274.64)	4.59(−2.96-12.62)	3.96(3.85-4.06)	0.14(0.13-0.15)
Taiwan (Province of China)	89537.18(73423.61-109882.74)	625.66(511.59-783.68)	8941.90(5809.29-12902.95)	62.15(40.41-88.83)	263228.52(213898.59-327992.81)	672.69(558.99-823.74)	25554.67(16385.50-36588.33)	65.23(42.19-92.48)	193.99(166.08-229.07)	7.52(−1.29-17.33)	3.43(3.35-3.52)	0.24(0.13-0.34)	185.79(158.30-218.71)	4.95(−4.52-14.82)	3.29(3.21-3.38)	0.12(0.02-0.22)
United Arab Emirates	11083.44(9611.33-12733.59)	795.56(676.99-937.90)	634.49(420.72-885.00)	74.11(48.00-103.23)	37719.25(32116.66-45001.31)	861.26(719.42-1024.69)	4287.02(2843.57-6069.15)	80.44(53.54-112.46)	573.24(494.88-660.13)	8.26(0.29-17.11)	6.70(6.39-7.02)	0.26(0.24-0.29)	575.67(481.99-679.28)	8.55(−0.51-17.41)	6.72(6.40-7.03)	0.27(0.25-0.29)
**High-middle SDI**																
Bahrain	1932.13(1677.89-2208.62)	751.16(633.17-903.24)	181.68(121.09-253.66)	70.06(46.08-98.37)	7969.23(6690.61-9423.58)	814.75(692.47-970.29)	754.84(515.38-1050.10)	76.67(51.13-106.72)	312.46(279.12-352.49)	8.47(1.52-17.46)	5.17(4.92-5.42)	0.29(0.28-0.30)	315.47(278.38-362.19)	9.44(1.68-18.68)	5.20(4.95-5.46)	0.32(0.31-0.33)
Georgia	40075.98(33409.43-49616.52)	707.12(590.67-868.02)	3798.34(2429.81-5390.26)	66.91(42.99-94.72)	39967.61(33016.77-49475.56)	714.52(604.95-864.68)	3719.39(2389.18-5320.30)	66.79(42.98-95.17)	−0.27(−8.76-10.03)	1.05(−6.73-9.57)	2.69(2.55-2.83)	0.03(NaN-NaN)	−2.08(−11.04-8.46)	−0.17(−8.64-9.71)	−0.07(NaN-NaN)	−0.01(NaN-NaN)
Israel	21936.64(18256.81-26503.41)	453.13(378.74-547.68)	2071.98(1357.04-2962.80)	42.72(28.43-60.89)	58944.58(49093.64-71445.51)	478.23(403.68-569.75)	5661.61(3642.64-7980.05)	45.98(30.21-63.89)	168.70(145.91-195.02)	5.54(−1.58-14.82)	3.13(3.05-3.21)	0.13(0.05-0.21)	173.25(147.87-202.99)	7.64(−0.60-17.78)	3.20(3.11-3.29)	0.21(0.13-0.30)
Jordan	15220.93(13307.14-17373.86)	777.02(653.37-937.85)	1451.40(990.10-2011.39)	73.27(48.53-102.95)	65573.51(56005.03-75716.49)	816.99(695.06-962.64)	6235.01(4152.79-8628.38)	77.04(51.06-106.42)	330.81(299.37-365.62)	5.14(−1.96-13.20)	4.95(4.65-5.25)	0.18(0.17-0.20)	329.59(292.03-368.45)	5.14(−2.33-14.24)	4.94(4.64-5.23)	0.18(0.16-0.20)
Kazakhstan	80991.85(68476.27-97428.07)	656.78(545.46-809.90)	7669.77(5057.95-10846.63)	62.08(40.84-88.71)	111376.39(94372.80-134019.83)	694.78(583.37-840.29)	10489.35(6810.73-14791.27)	65.35(42.18-92.74)	37.52(27.40-48.08)	5.79(−2.10-14.35)	1.19(0.96-1.42)	0.29(0.23-0.34)	36.76(25.46-49.98)	5.26(−3.66-14.94)	1.17(0.93-1.41)	0.28(0.22-0.34)
Lebanon	17183.29(14705.72-20264.04)	769.58(647.08-928.21)	1636.01(1078.96-2272.26)	72.94(47.78-102.72)	48761.60(40822.78-58603.89)	812.75(685.34-966.24)	4643.05(3039.03-6487.59)	77.52(50.67-108.24)	183.77(161.80-208.42)	5.61(−2.39-13.17)	3.65(3.59-3.72)	0.19(0.18-0.21)	183.80(160.51-208.53)	6.27(−2.10-14.05)	3.65(3.58-3.72)	0.21(0.19-0.23)
Malaysia	60613.78(52349.80-69864.20)	562.22(467.68-680.71)	5791.12(3850.04-8052.66)	53.73(34.86-76.41)	157560.80(132837.34-187767.69)	607.56(512.28-729.19)	15208.85(9872.81-21403.30)	58.43(38.27-81.69)	159.94(136.26-184.10)	8.07(−0.05-17.66)	3.08(3.02-3.14)	0.26(0.25-0.27)	162.62(137.35-188.19)	8.73(−0.34-19.16)	3.12(3.06-3.17)	0.28(0.27-0.29)
Oman	7432.50(6578.11-8375.92)	692.49(594.04-807.42)	695.13(476.04-970.03)	64.01(43.09-88.67)	20007.87(17167.03-23302.75)	762.38(641.21-921.08)	1877.30(1261.80-2593.37)	70.77(47.45-99.42)	169.19(149.49-193.40)	10.09(2.55-20.96)	2.97(2.65-3.30)	0.31(0.29-0.33)	170.07(146.29-198.10)	10.56(1.79-21.65)	2.98(2.65-3.31)	0.32(0.30-0.34)
Saudi Arabia	62837.84(54831.34-71281.63)	742.10(621.90-893.67)	5940.41(4002.52-8303.91)	69.35(45.61-96.85)	166148.12(143790.96-193176.45)	802.96(678.58-961.03)	15777.51(10323.62-21884.05)	75.63(49.48-105.87)	164.41(141.17-189.48)	8.20(0.28-16.69)	3.07(2.97-3.16)	0.27(0.26-0.28)	165.60(140.25-194.89)	9.06(0.31-18.45)	3.09(2.99-3.18)	0.30(0.29-0.31)
Sri Lanka	61489.08(52409.95-72627.75)	569.70(471.53-690.28)	5875.84(3863.62-8222.60)	54.40(35.28-76.19)	148884.88(124476.02-184152.83)	613.63(515.76-742.30)	14325.98(9255.81-20507.31)	59.00(38.34-84.77)	142.13(121.18-164.44)	7.71(−0.66-15.80)	2.83(2.75-2.90)	0.24(0.23-0.26)	143.81(121.03-166.47)	8.45(−0.46-17.18)	2.87(2.79-2.94)	0.29(0.27-0.30)
**Middle SDI**																
Armenia	18745.32(15788.11-22425.49)	721.25(591.42-875.25)	1749.73(1151.94-2433.61)	67.11(43.15-93.96)	30353.46(25727.81-36285.07)	769.96(660.84-902.72)	2818.54(1820.41-3919.43)	71.62(47.43-98.98)	61.93(46.70-80.75)	6.75(−4.75-18.96)	1.80(1.71-1.90)	0.32(0.26-0.39)	61.08(45.58-79.94)	6.71(−4.57-19.09)	1.77(1.68-1.87)	0.32(0.26-0.38)
Azerbaijan	33106.98(28173.31-39184.23)	653.75(542.28-796.41)	3092.22(2048.10-4416.99)	60.79(39.52-86.93)	58116.67(49031.55-69397.00)	685.59(577.86-828.31)	5426.20(3537.93-7677.44)	63.81(41.77-91.03)	75.54(61.54-90.38)	4.87(−3.38-14.97)	1.85(1.80-1.90)	0.22(0.18-0.27)	75.48(60.41-91.97)	4.95(−3.97-14.96)	1.84(1.79-1.90)	0.23(0.18-0.27)
China	4683302.58(4036498.77-5435212.21)	644.58(558.65-751.14)	459519.87(313552.36-630784.81)	62.83(42.81-87.18)	13099726.59(11320895.05-15376466.89)	692.50(607.26-802.00)	1290809.65(865893.96-1775731.18)	67.79(45.92-93.34)	179.71(167.15-191.87)	7.44(2.75-11.66)	3.35(3.28-3.43)	0.23(0.19-0.27)	180.90(167.50-194.17)	7.91(3.18-12.22)	3.36(3.30-3.42)	0.24(0.20-0.28)
Indonesia	506389.92(450318.18-568265.41)	512.47(445.76-591.20)	50573.44(34714.40-69096.65)	50.66(34.73-71.12)	1099460.03(963117.28-1264038.28)	543.69(475.80-623.55)	109257.76(73518.55-150497.39)	53.75(36.33-74.86)	117.12(108.21-126.13)	6.09(2.86-9.71)	2.41(2.37-2.46)	0.17(0.16-0.19)	116.04(106.39-126.10)	6.09(2.14-10.19)	2.40(2.35-2.44)	0.17(0.16-0.19)
Iran (Islamic Republic of)	254230.65(229905.66-282073.15)	774.54(683.84-877.14)	23918.83(16350.95-33261.37)	72.21(49.55-99.83)	600289.09(533023.47-675047.66)	809.51(717.15-921.19)	56127.34(38638.75-76380.73)	75.52(52.12-103.73)	136.12(123.30-150.62)	4.52(1.53-7.60)	2.94(2.89-2.99)	0.13(0.12-0.14)	134.66(121.34-148.39)	4.57(1.48-7.72)	2.91(2.86-2.96)	0.13(0.12-0.14)
Iraq	80024.44(69019.74-91699.93)	723.18(599.87-868.82)	7582.53(5050.25-10638.40)	68.28(44.53-96.40)	198109.92(170051.36-229127.05)	752.72(640.94-906.68)	18820.16(12468.49-26161.59)	71.18(47.42-99.06)	147.56(131.68-165.57)	4.08(−2.80-12.15)	3.11(3.06-3.17)	0.15(0.14-0.16)	148.20(129.07-168.53)	4.25(−3.11-12.46)	3.12(3.07-3.17)	0.15(0.14-0.17)
Philippines	180440.53(161393.44-202032.79)	541.36(472.74-624.76)	17243.30(11772.87-23739.32)	51.23(34.83-71.41)	429778.93(382305.29-488254.85)	554.20(488.33-636.83)	40971.55(28166.28-56498.41)	52.50(35.78-73.60)	138.18(130.01-146.85)	2.37(−0.17-4.86)	2.80(2.78-2.82)	0.06(0.05-0.07)	137.61(128.74-146.29)	2.47(−0.17-5.18)	2.80(2.78-2.82)	0.06(0.05-0.07)
Syrian Arab Republic	54080.44(46645.10-61927.07)	750.52(620.40-893.95)	5124.00(3497.25-7119.18)	70.01(45.96-97.97)	93997.87(78984.11-113264.12)	791.02(664.75-960.92)	8830.47(5769.64-12367.08)	73.88(48.77-103.31)	73.81(56.82-91.71)	5.40(−2.72-13.16)	1.97(1.63-2.31)	0.18(0.15-0.22)	72.34(53.59-91.58)	5.53(−2.76-13.83)	1.93(1.59-2.27)	0.18(0.15-0.22)
Thailand	198340.11(167075.68-235406.86)	577.62(471.84-702.42)	20029.45(13116.74-28445.62)	58.10(37.50-83.28)	608167.26(495441.56-751305.28)	625.22(524.14-761.67)	61731.69(39162.25-87420.14)	63.29(40.54-89.51)	206.63(173.82-241.65)	8.24(0.33-17.43)	3.70(3.61-3.78)	0.25(0.25-0.26)	208.20(174.42-250.24)	8.93(0.16-19.34)	3.72(3.63-3.80)	0.28(0.27-0.28)
Turkmenistan	14364.77(12204.55-16690.77)	658.47(541.16-806.95)	1346.87(912.73-1886.45)	61.18(40.06-86.41)	25147.40(21553.55-29558.59)	676.78(569.83-817.86)	2380.12(1552.58-3358.21)	63.69(41.37-90.22)	75.06(60.53-88.44)	2.78(−5.43-11.51)	1.85(1.76-1.93)	0.15(0.11-0.19)	76.71(61.32-91.91)	4.10(−4.58-13.47)	1.88(1.79-1.96)	0.20(0.15-0.24)
Uzbekistan	87985.19(75680.34-103040.20)	638.54(530.28-794.02)	8403.49(5526.15-11654.05)	60.41(39.40-87.10)	159099.88(135661.06-187255.84)	663.55(553.96-813.78)	14974.75(9912.58-21423.12)	61.99(40.12-88.87)	80.83(67.11-94.78)	3.92(−4.58-13.18)	1.86(1.72-1.99)	0.18(0.14-0.23)	78.20(62.02-94.15)	2.61(−6.50-12.81)	1.78(1.67-1.90)	0.12(0.08-0.16)
Viet Nam	235316.26(199946.96-280328.94)	533.71(440.32-655.49)	23667.60(15933.68-32904.02)	53.61(35.68-75.73)	513271.71(430942.02-627683.65)	586.76(487.87-718.85)	51876.94(33818.32-72985.93)	59.17(37.96-82.50)	118.12(99.90-139.15)	9.94(0.88-19.23)	2.48(2.45-2.51)	0.30(0.29-0.31)	119.19(98.16-141.20)	10.37(0.64-20.80)	2.49(2.47-2.52)	0.32(0.31-0.32)
**Low-middle SDI**																
Bangladesh	336392.09(291090.88-390595.25)	540.54(453.90-648.21)	32617.99(22500.18-45336.68)	52.04(34.45-72.68)	809357.76(685010.34-963193.91)	602.95(510.28-719.33)	78324.78(51079.41-107523.13)	58.20(38.28-79.97)	140.60(118.59-161.47)	11.55(2.49-20.31)	2.98(2.81-3.15)	0.38(0.37-0.40)	140.13(115.95-164.49)	11.83(2.49-21.83)	2.96(2.79-3.12)	0.38(0.36-0.39)
Bhutan	1735.57(1493.84-1987.64)	535.92(452.04-639.88)	163.81(113.07-231.04)	50.20(32.93-70.78)	3674.02(3126.65-4298.80)	604.69(515.63-713.75)	346.00(227.49-484.44)	56.74(37.13-78.96)	111.69(92.36-132.47)	12.83(5.68-21.70)	2.66(2.58-2.75)	0.43(0.41-0.44)	111.22(90.00-134.25)	13.03(4.62-22.56)	2.65(2.56-2.73)	0.43(0.42-0.44)
Cambodia	24705.81(20940.44-28769.42)	480.76(391.12-599.51)	2408.28(1610.24-3402.42)	46.69(30.29-66.12)	58856.86(49295.43-70911.28)	520.24(430.79-644.82)	5768.83(3752.13-8123.33)	50.75(33.28-73.13)	138.23(120.00-158.72)	8.21(−0.30-18.40)	2.85(2.78-2.93)	0.27(0.25-0.29)	139.54(119.02-163.46)	8.70(−0.51-19.75)	2.87(2.80-2.94)	0.28(0.27-0.30)
Democratic People’s Republic of Korea	75072.95(62107.62-91697.88)	542.33(440.94-677.83)	7357.96(4781.24-10363.33)	52.96(34.41-75.60)	171489.34(140045.74-214279.88)	595.34(492.56-731.51)	16853.63(10924.28-23869.07)	58.18(37.92-82.88)	128.43(108.42-151.93)	9.77(0.90-19.40)	2.80(2.73-2.87)	0.31(0.29-0.32)	129.05(106.95-153.68)	9.86(0.20-21.15)	2.81(2.74-2.87)	0.30(0.29-0.32)
India	2715763.24(2448378.61-3016348.95)	532.40(471.71-605.64)	253150.47(172879.96-347081.39)	48.98(33.50-66.79)	6722227.99(5937217.45-7631762.23)	595.16(526.69-677.85)	624997.28(425464.17-854741.44)	54.96(37.44-75.83)	147.53(136.71-158.64)	11.79(9.73-14.03)	3.12(3.08-3.16)	0.42(0.40-0.44)	146.89(135.31-158.60)	12.21(9.71-14.85)	3.11(3.06-3.15)	0.43(0.41-0.45)
Kyrgyzstan	21626.06(18536.33-25953.97)	675.87(560.92-839.34)	2079.98(1377.36-2936.10)	64.85(42.93-91.72)	32591.52(27673.79-38596.64)	697.19(581.54-843.99)	3077.00(2020.50-4341.67)	65.31(42.44-93.25)	50.70(40.86-62.00)	3.15(−4.26-11.96)	1.25(1.13-1.36)	0.16(0.12-0.21)	47.93(37.21-60.04)	0.70(−6.93-9.04)	1.17(1.06-1.27)	0.07(0.02-0.11)
Lao People’s Democratic Republic	10362.93(8713.67-12175.11)	473.84(389.84-585.40)	998.67(651.92-1402.46)	45.48(29.22-64.37)	22533.64(19056.62-26892.29)	513.59(425.69-630.29)	2180.23(1427.63-3086.05)	49.49(32.29-70.80)	117.44(102.53-134.17)	8.39(−0.21-17.74)	2.42(2.38-2.47)	0.27(0.25-0.29)	118.31(99.45-138.94)	8.83(−0.53-19.24)	2.43(2.39-2.47)	0.28(0.26-0.30)
Maldives	593.63(511.72-685.59)	556.77(463.16-683.24)	57.93(38.99-80.59)	54.45(35.37-78.06)	1975.11(1698.14-2308.19)	610.49(513.09-738.67)	193.16(129.63-268.91)	59.60(39.12-83.63)	232.72(201.90-265.89)	9.65(0.50-19.88)	3.97(3.90-4.03)	0.31(0.30-0.33)	233.47(198.94-267.49)	9.45(0.06-19.21)	3.97(3.91-4.02)	0.30(0.29-0.31)
Mongolia	7863.95(6744.11-9260.94)	620.90(518.98-769.35)	752.55(505.64-1064.07)	58.82(38.27-83.99)	13717.25(11705.74-16203.24)	645.43(542.86-787.00)	1307.97(861.06-1869.80)	61.22(39.76-87.86)	74.43(60.25-89.20)	3.95(−5.91-12.51)	1.84(1.55-2.13)	0.22(0.17-0.26)	73.81(59.02-90.13)	4.08(−6.64-14.68)	1.83(1.55-2.11)	0.22(0.18-0.27)
Myanmar	109860.55(91666.10-131881.83)	488.89(403.00-600.89)	10602.03(6944.50-14862.39)	46.78(30.53-65.93)	229237.29(191128.24-279783.05)	523.75(431.84-642.52)	22169.01(14210.33-31774.80)	50.40(32.59-72.50)	108.66(90.79-129.92)	7.13(−1.58-17.73)	2.38(2.34-2.43)	0.25(0.22-0.27)	109.10(88.95-131.37)	7.72(−1.81-19.17)	2.39(2.35-2.43)	0.27(0.25-0.29)
Palestine	9129.91(7925.38-10504.23)	729.86(611.57-865.47)	865.56(581.64-1215.85)	68.73(44.92-95.76)	23904.46(20804.41-27494.80)	760.54(641.97-905.18)	2274.06(1511.34-3177.84)	71.91(46.87-99.46)	161.83(145.53-178.42)	4.20(−2.62-11.55)	3.19(3.12-3.25)	0.12(0.11-0.13)	162.73(142.15-183.69)	4.63(−2.84-11.89)	3.20(3.13-3.27)	0.14(0.13-0.15)
Tajikistan	21361.07(18160.64-25114.00)	649.90(532.35-793.54)	2034.42(1352.08-2860.34)	61.12(39.34-86.08)	39081.13(33587.73-45518.00)	666.05(557.13-818.61)	3726.45(2484.49-5208.68)	62.61(40.89-89.04)	82.95(71.12-96.63)	2.49(−5.46-12.05)	1.93(1.76-2.10)	0.13(0.08-0.17)	83.17(68.63-99.10)	2.45(−6.21-12.62)	1.93(1.76-2.09)	0.12(0.08-0.17)
Timor-Leste	1670.41(1438.76-1914.62)	505.35(417.54-620.91)	160.34(108.05-223.71)	48.18(30.99-67.14)	4781.83(4053.96-5714.17)	533.05(445.12-657.90)	460.30(303.21-638.77)	51.05(33.31-71.79)	186.27(159.93-215.03)	5.48(−3.47-14.78)	3.66(3.57-3.74)	0.18(0.17-0.19)	187.08(157.35-218.75)	5.96(−3.25-15.44)	3.67(3.58-3.75)	0.19(0.18-0.20)
**Low SDI**																
Afghanistan	43545.24(36511.93-51687.66)	623.74(516.69-761.11)	4084.55(2693.42-5658.64)	58.02(38.22-82.01)	94872.42(82453.84-108119.83)	628.56(527.04-759.26)	8981.60(6059.42-12608.58)	58.67(38.33-81.89)	117.87(97.41-142.28)	0.77(−7.34-9.13)	2.47(2.40-2.55)	0.03(0.02-0.05)	119.89(97.06-146.90)	1.12(−7.14-10.02)	2.50(2.42-2.58)	0.04(0.02-0.06)
Nepal	57370.74(49168.04-66047.02)	519.93(429.23-618.35)	5390.31(3591.37-7544.37)	48.26(31.56-67.86)	134577.17(112915.47-158903.66)	583.58(487.67-696.96)	12587.34(8315.99-17419.78)	54.22(35.98-74.81)	134.57(117.32-154.40)	12.24(4.08-21.87)	2.88(2.84-2.91)	0.40(0.39-0.40)	133.52(113.85-157.65)	12.34(3.90-22.32)	2.85(2.82-2.89)	0.39(0.38-0.40)
Pakistan	374279.17(332523.11-416453.70)	543.29(479.67-620.59)	35085.89(23541.79-48299.92)	50.42(34.16-69.94)	803961.95(719442.92-893678.28)	577.37(507.37-661.31)	75368.95(51162.24-103772.27)	53.49(36.24-74.10)	114.80(107.17-123.25)	6.27(1.71-10.64)	2.50(2.42-2.59)	0.21(0.20-0.23)	114.81(104.95-125.05)	6.09(1.03-10.72)	2.50(2.42-2.59)	0.21(0.19-0.22)
Yemen	46402.82(40057.48-53629.43)	657.75(550.11-789.27)	4350.51(2937.08-6115.50)	61.16(40.16-85.72)	125562.87(108471.57-143823.99)	683.02(579.74-818.08)	11752.24(7896.29-16362.07)	63.55(41.08-88.99)	170.59(154.78-187.72)	3.84(−3.57-12.41)	3.38(3.33-3.42)	0.14(0.13-0.16)	170.13(148.42-194.83)	3.90(−4.15-13.57)	3.36(3.32-3.41)	0.14(0.12-0.16)
**Disease Types**																
Treated heart failure	4261483.92(3706767.66-4932191.58)	214.42(184.60-252.51)	278834.63(189378.34-384099.45)	14.22(9.71-19.73)	10854342.59(9330395.95-12706946.73)	232.82(200.59-271.89)	722729.28(482395.60-1004827.26)	15.39(10.37-21.44)	154.71(145.66-164.04)	8.58(5.68-11.38)	3.08(3.04-3.13)	0.26(0.24-0.29)	159.20(147.44-170.99)	8.17(3.92-12.01)	3.11(3.09-3.13)	0.22(0.19-0.25)
Mild heart failure	2162679.55(1681679.60-2744898.54)	108.76(82.46-143.47)	87932.53(51607.16-138803.22)	4.39(2.54-7.02)	5507018.96(4162321.87-7193390.08)	118.13(89.48-154.02)	223086.46(128608.21-359744.28)	4.78(2.77-7.70)	154.64(143.89-164.59)	8.61(5.60-11.48)	3.08(3.04-3.13)	0.26(0.24-0.29)	153.70(142.88-164.30)	8.87(5.92-11.86)	1.82(1.77-1.88)	0.27(0.25-0.30)
Moderate heart failure	1398784.79(1058757.54-1797616.56)	70.36(51.90-91.47)	97984.30(61154.08-150528.65)	4.89(3.06-7.46)	3562061.98(2636509.19-4644178.52)	76.40(57.10-98.83)	248575.86(154629.92-380216.36)	5.33(3.34-8.11)	154.65(143.42-165.26)	8.59(5.67-11.39)	1.83(1.78-1.89)	0.26(0.24-0.29)	153.69(142.08-164.34)	8.99(6.06-12.04)	1.82(1.76-1.88)	0.28(0.25-0.30)
Severe heart failure	3778045.225(3239467.907-4412417.382)	190.09(160.44-226.62)	654854.169(433571.942-935297.249)	32.61(21.74-46.90)	9623155.132(8176531.522-11389810.974)	206.41(176.78-244.07)	1661319.401(1089763.715-2386524.585)	35.59(23.42-51.06)	154.71(145.63-163.82)	8.59(5.71-11.39)	1.83(1.78-1.89)	0.26(0.24-0.29)	153.69(144.55-163.11)	9.14(6.07-11.96)	1.82(1.77-1.88)	0.28(0.26-0.31)

There was considerable variation in the number of HF cases across countries and regions in 2021. Among the 48 Asian countries and regions, China had the highest number of HF cases, with 13.10 million cases (95% UI: 11.32 million-15.38 million), reflecting a 179.71% (95% UI: 167.15–191.87%) increase, and an EAPC-Count of 3.35% (95% CI: 3.28–3.43%). Similarly, in 2021, Taiwan’s age-standardized prevalence rate was 672.69 per 100,000 population (95% UI: 558.99–823.74), showing a 193.99% increase (95% UI: 166.08–229.07), with an EAPC-ASR of 3.43% (95% CI: 3.35–3.52).

**Fig 1:**
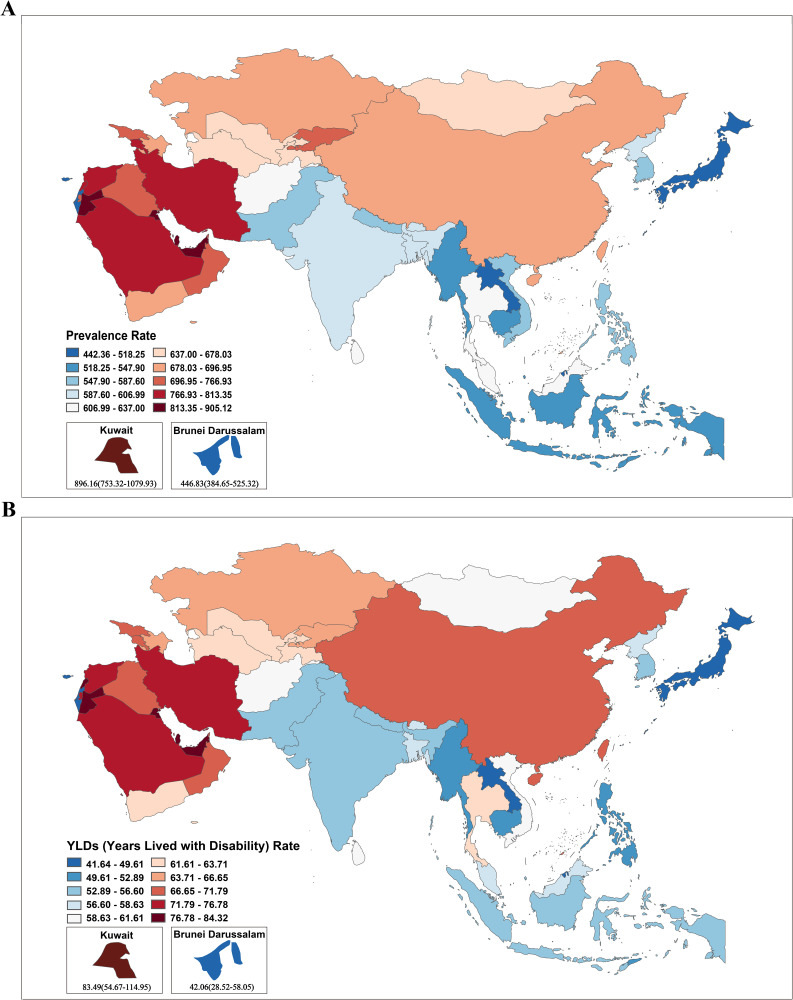
Map distribution of age-standardized prevalence (A) and YLD (B) rates for HF in 48 Asian countries, 2021.

From 1990 to 2021, Bhutan experienced the largest increase in age-standardized prevalence rate, with a growth of 12.83% (95% UI: 5.68–21.70), while Japan saw the largest decrease in age-standardized prevalence rate, with a decline of 2.72% (95% UI: −7.52 to 2.55) (Table 1 and Figs 1 and S1).

### 3.2 Trends in YLDs of HF in Asia and by country

Similar to the trend in prevalence, the number of YLDs attributable to HF in Asia has also increased by 155.06% (95% UI: 145.93–164.43%) from 1990 to 2021, rising from 1.12 million (95% UI: 0.76 million-1.53 million) in 1990 to 2.86 million (95% UI: 1.94 million-3.97 million) in 2021. The age-standardized YLD rate attributable to HF increased from 56.11 (95% UI: 38.46–77.56) per 100,000 population in 1990 to 61.08 per 100,000 population (95% UI: 41.60–84.91) in 2021.

As shown in Table 1 and Fig 1, China had the highest number of HF-related YLDs, increasing from 0.45 million (95% UI: 0.31 million-0.63 million) in 1990 to 1.29 million (95% UI: 0.87 million-1.78 million) in 2021.

In contrast, Brunei Darussalam had the lowest age-standardized YLD rate in 2021, with a rate of 42.06 per 100,000 population (95% UI: 28.52–58.05) (Table 1 and Fig 1).

### 3.3 Trends in prevalence and YLDs by HF severity across Asia

**Fig 2:**
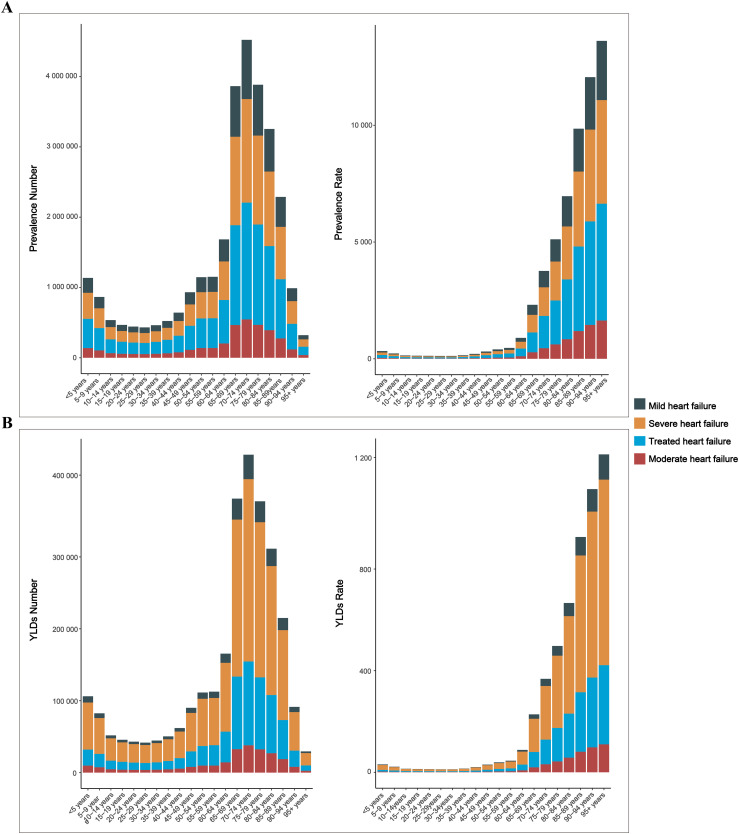
Distribution of prevalence numbers, prevalence rates (A), YLDs numbers, and YLD rates (B) for four types of HF severity by age group in Asia, 2021.

Based on the classification of HF severity, in 2021, the highest number of HF cases in Asia was observed for treated HF [10.85 million(95% UI: 9.33 million-12.71 million)], followed by severe HF [9.62 million (95% UI: 8.18 million-11.39 million)], mild HF [5.51 million (95% UI: 4.16–7.19 million)], and moderate HF [3.56 million (95% UI: 2.63 million-4.64 million)]. (Fig 2)

From 1990 to 2021, the age-standardized prevalence rate of mild HF showed the greatest increase, rising by 8.61% (95% UI: 5.60–11.48%), while treated HF had the smallest increase in age-standardized prevalence rate, with a rise of 8.58% (95% UI: 5.68–11.38%). In terms of YLDs, severe HF exhibited the highest increase in age-standardized YLD rate, with a growth of 9.14% (95% UI: 6.07–11.96%), while treated HF had the lowest increase in age-standardized YLD rate, rising by 8.17% (95% UI: 3.92–12.01%) (Table 1).

### 3.4 Trends in prevalence and YLDs of HF by sex and age group across Asia

From 1990 to 2021, the overall crude prevalence and crude YLD rate in the Asian population showed a continuous increasing trend, with the rate of increase gradually accelerating over time. In contrast, the age-standardized prevalence and YLD

rates remained relatively stable, although considerable variation observed across different age groups.

**Fig 3:**
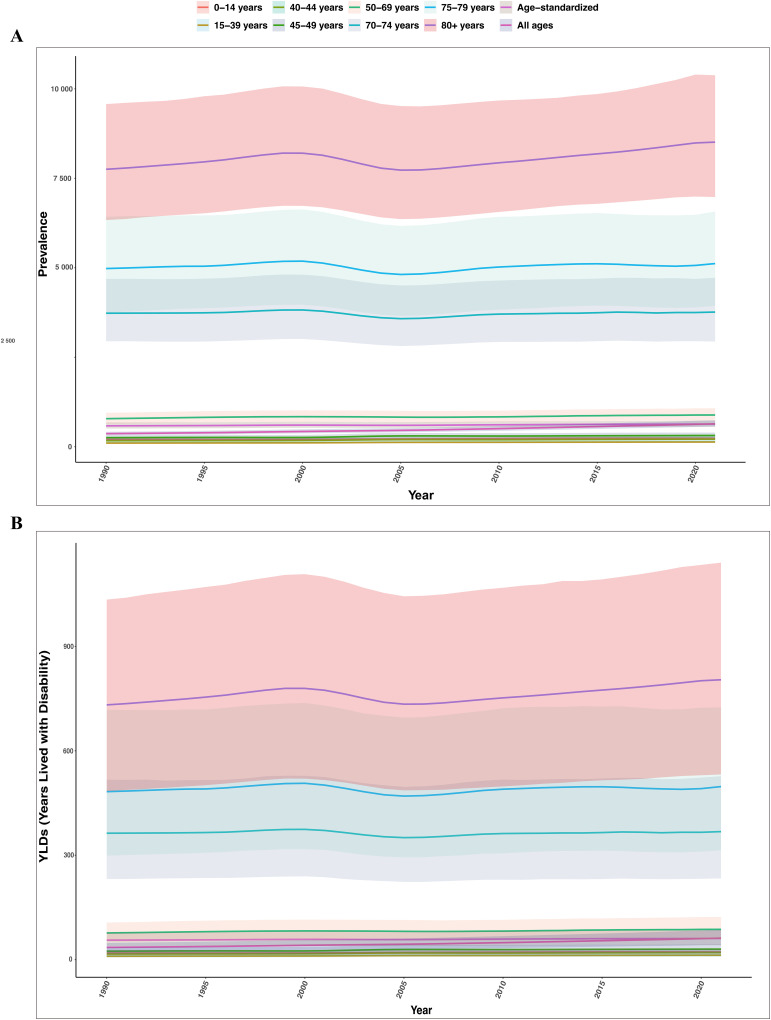
Trend of prevalence rate (A) and YLD rate (B) for HF by age group in Asia, 1990 to 2021.

**Fig 4:**
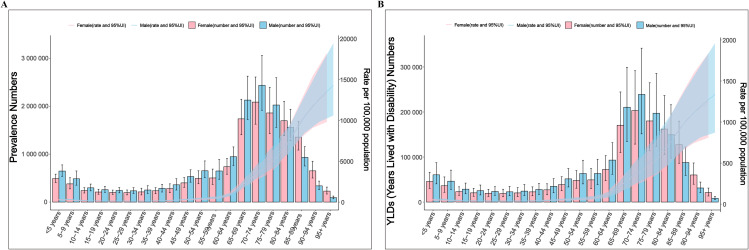
Distribution of HF prevalence numbers, prevalence rates (A), YLDs numbers, and YLD rates (B) across age groups in Asia, 2021.

Over this 32-year period, the prevalence of HF increased steadily with advancing age. The age-standardized prevalence rate was generally higher in men than in women. In 2021, the number of HF cases among men peaked in the 70−74 age group [2.44 million (95% UI: 1.92 million-3.06 million)], closely followed by the number among women in the same age group [2.09 million (95% UI: 1.63 million-2.61 million)] (Figs 3 and 4 and S1 Table).

As age increased, the highest prevalence rates were observed in the population aged 95 years and older, with males at 14,289.22 per 100,000 population (95% UI: 10,589.26–19,424.85) and females at 13,359.62 per 100,000 population (95% UI: 9,811.63–18,239.38). Regarding the distribution by HF severity, the prevalence and YLDs for all severity classifications increased significantly with age. This increase was particularly sharp for prevalence among individuals aged 60 years and older. In 2021, treated HF had the highest prevalence rate [232.82 per 100,000 population (95% UI: 200.59–271.89)], while moderate HF had the lowest[76.40 per 100,000 population (95% UI: 57.10–98.83)].

In terms of YLDs, severe HF accounted for the highest number of YLDs [1.66million (95% UI: 1.09 million-2.39 million)], whereas mild HF accounted for the lowest [0.22 million (95% UI: 0.13 million-0.36 million)]. Notably, severe HF also had the highest YLD rate [35.59 per 100,000 population (95% CI: 23.42–51.06)]. Among the elderly (aged 80 years and older), severe HF showed a significantly higher prevalence compared to other severity classifications, indicating a greater burden of advanced HF in this population (Fig 4 and S2 Table).

### 3.5 Trends in the distribution of HF prevalence and YLDs across different SDI regions

The results indicate that from 1990 to 2021, both the prevalence and YLD rate of HF showed an upward trend across all SDI regions in Asia. High SDI regions generally exhibited higher prevalence and YLD rate compared to other SDI regions, while the increase in both prevalence and YLD rate was relatively smaller in low SDI regions.

#### 3.5.1 High SDI regions.

In 2021, Kuwait recorded the highest prevalence and YLD rates, at 896.16 per 100,000 population (95% UI: 753.32–1079.93) and 83.49 per 100,000 population (95% UI: 54.67–114.95), respectively. In contrast, Brunei Darussalam had the lowest rates, with a prevalence rate of 446.83 per 100,000 population (95% UI: 384.65–525.32) and a YLD rate of 42.06 per 100,000 population (95% UI: 28.52–58.05)

**Fig 5:**
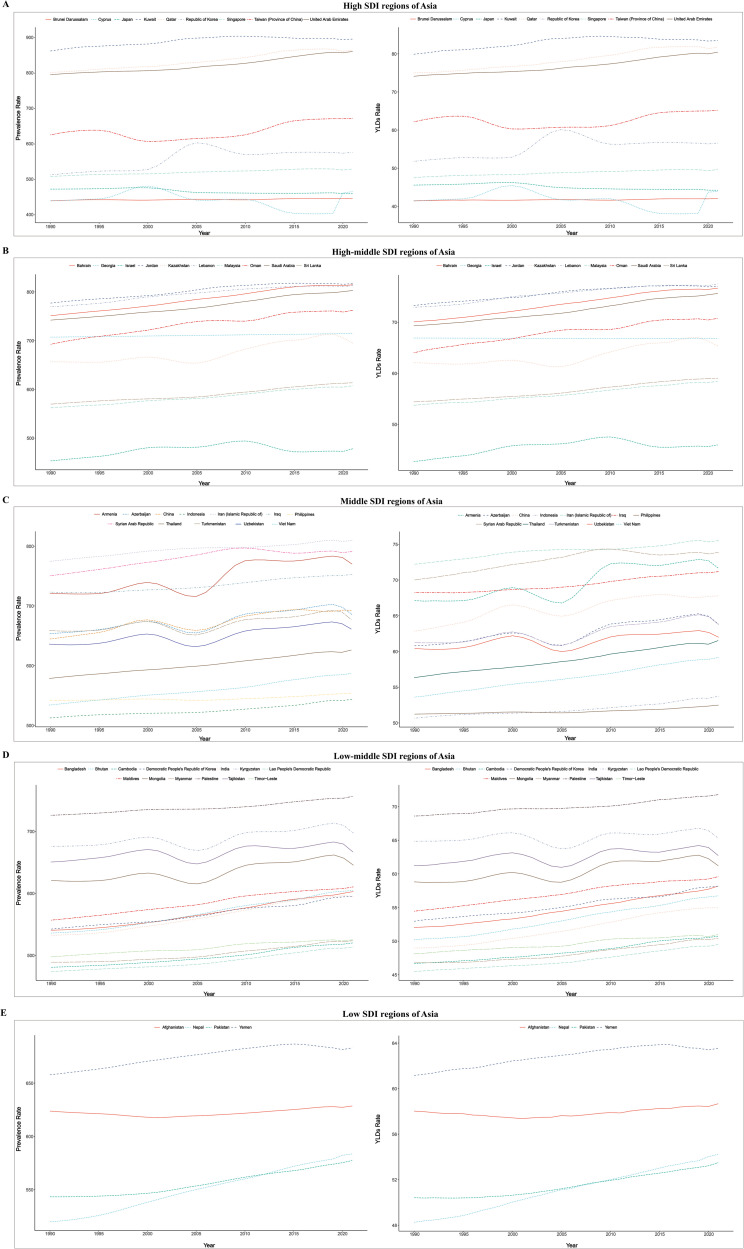
Trend of age-standardized prevalence and YLD rate distribution in different SDI regions across 48 Asian countries, 1990 to 2021.

The largest increases in both prevalence and YLD rates from 1990 to 2021 were observed in the Republic of Korea, with an increase of 12.40% (95% UI: 3.67–21.15) and 9.26% (95% UI: −0.69–19.07), respectively. In contrast, Japan showed the smallest changes, with a decline in prevalence of 2.72% (95% UI: −7.52 to 2.55) and a decline in YLDs of 3.00% (95% UI: −8.16 to 3.13).(Fig 5A).

#### 3.5.2 High-middle SDI regions.

In 2021, Jordan had the highest prevalence rate, at 816.99 per 100,000 population (95% UI: 695.06–962.64), while Lebanon had the highest YLD rate at 77.52 per 100,000 population (95% UI: 50.67–108.24).In contrast, Israel reported the lowest rates: a prevalence rate of 478.23 per 100,000 population (95% UI: 403.68–569.75) and a YLD rate of 45.98 per 100,000 population (95% UI: 30.21–63.89).

Oman experienced the largest increase in both prevalence and YLD rate, with prevalence rate rising from 692.49 per 100,000 population in 1990 to 762.38 per 100,000 population in 2021, reflecting an increase of 10.09% (95% UI: 2.55%−20.96%). Similarly, the YLD rate increased by 10.56% (95% UI: 1.79%−21.65%), rising from 64.01 to 70.77 per 100,000 population.

Georgia showed the smallest changes over this period. The prevalence rate increased by 1.05% (95% UI: −6.73 to 9.57), from 707.12 to 714.52 per 100,000 population, while the YLD rate remained relatively stable, with a change of −0.17% (95% UI: −8.64 to 9.71) (Fig 5B).

#### 3.5.3 Middle SDI regions.

In 2021, Iran had the highest prevalence rate at 809.51 per 100,000 population (95% UI: 717.15–921.19) and the highest YLD rate at 75.52 per 100,000 population (95% UI: 52.12–103.73). In contrast, Indonesia reported the lowest prevalence rate [543.69 per 100,000 population (95% UI: 475.80–623.55)], while the Philippines had the lowest YLD rate (52.50 per 100,000 population (95% UI: 40.54–89.51)].

Vietnam experienced the largest increases in both prevalence [9.94% (95% UI: 0.88–19.23%)] and YLD rate [10.37% (95% UI: 0.64–20.80%)].

Conversely, the Philippines showed the smallest relative changes over this period. The prevalence rate increased by only 2.37% (95% UI: −0.17–4.86), and the YLD rate increased by 2.47% (95% UI: −0.17 to 5.18) (Fig 5C).

#### 3.5.4 Low-middle SDI regions.

Palestine had the highest prevalence rate at 760.54 per 100,000 population (95% UI: 641.97–905.18) and the highest YLD rate at 71.91 per 100,000 population (95% UI: 46.87–99.46) in 2021. In contrast, the Lao People’s Democratic Republic had the lowest rates: a prevalence rate of 513.59 per 100,000 population (95% UI: 425.69–630.29) and a YLD rate of 49.49 per 100,000 population (95% UI: 32.29–70.80).

Bhutan showed the largest relative increases from 1990 to 2021. The prevalence rate increased by 12.83% (95% UI: 5.68–21.70), and the YLD rate increased by 13.03% (95% UI: 4.62–22.56).

Conversely, Tajikistan showed the smallest relative changes over this period. The prevalence rate increased by only 2.49% (95% UI: −5.46 to 12.05), and the YLD rate increased by 2.45% (95% UI: −6.21 to 12.62) (Fig 5D).

#### 3.5.5 Low SDI regions.

In 2021, Yemen had the highest prevalence rate, at 683.02 per 100,000 population (95% UI: 579.74–818.08), and the highest YLD rate, at 63.55 per 100,000 population (95% UI: 41.08–88.99). Yemen’s prevalence rate increased by 3.84% (95% UI: −3.57%−12.41%) from 657.75 in 1990 to 683.02 per 100,000 population in 2021, while its YLD rate increased by 3.90% (95% UI: −4.15 to 13.57%).

Pakistan had the lowest prevalence rate [577.37 per 100,000 population (95% UI: 507.37–661.31)] and YLD rate [53.49 per 100,000 population (95% UI: 36.24–74.10)].

Nepal experienced the largest increases in both prevalence [12.24% (95% UI: 4.08–21.87%)] and YLD rate [12.34% (95% UI: 3.90–22.32%)].

Conversely, Afghanistan showed the smallest relative changes, with a minimal increase in prevalence rate [0.77% (95% UI: −7.34 to 9.13%)] and YLD rate [1.12% (95% UI: −7.14 to 10.02%)] (Fig 5E).

#### 3.5.6 Correlation between SDI and trends in HF prevalence and YLDs.

**Fig 6:**
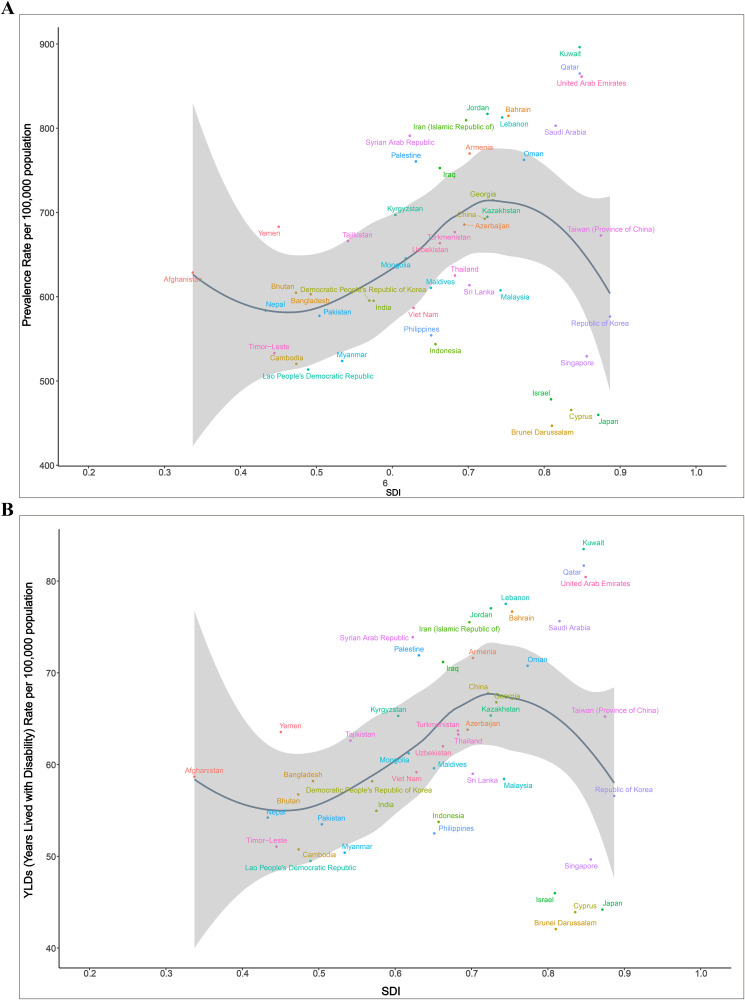
Distribution of age-standardized prevalence (A) and YLD rates (B) by SDI in 48 Asian countries, 2021.

The correlation between the EAPC-ASR in HF prevalence and SDI was weak, with R = 0.21, P = 0.16, and for YLDs, R = 0.23, p = 0.11. This suggests that SDI has a limited impact on the temporal trends of HF prevalence and YLD rates. The varying trends across SDI regions indicate that other factors, such as population aging, lifestyle changes, and healthcare resources, may play a more significant role in influencing the burden of HF (Figs 6 and S2).

### 3.6 Attribution analysis of HF in Asia

**Fig 7:**
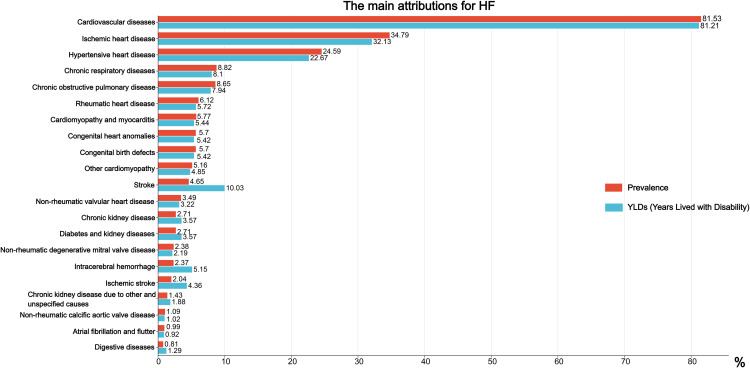
Distribution of major attributable causes for HF prevalence and YLDs in Asia, 2021.

In 2021, a total of 55 etiologies contributed to HF in Asia. Among these, cardiovascular diseases accounted for 81.53% of the prevalence and 81.21% of the YLDs attributable to HF. The primary causes of HF prevalence and YLDs were largely consistent, with the main contributors including ischemic heart disease, hypertensive heart disease, chronic respiratory diseases, chronic obstructive pulmonary disease, rheumatic heart disease, cardiomyopathy and myocarditis, congenital heart anomalies, congenital birth defects, other cardiomyopathies, stroke, non-rheumatic valvular heart disease, and chronic kidney disease (Fig 7 and S3 Table).

## 4. Discussion

Over the past 32 years, the prevalence of HF has greatly increased in Asia, becoming a major public health problem. Here, we investigated HF prevalence, and HF-associated YLDs among all-age population in Asia regions and countries from 1990 to 2021. The burden of HF in Asia remains notably high and males experiencing a higher burden than females. The overall burden of HF in Asia is lower than the global average. However, countries such as Kuwait, Qatar, and the United Arab Emirates, exhibit a significantly higher burden than the global level. In addition, over the past 30 years, Asia’s EAPC exceeded the global average, indicating a faster increase in the burden of HF. The relationship between the burden of HF and SDI generally shows an inverted U-shaped pattern, with the highest burden observed in high-middle SDI locations. No clear correlation was found between EAPC and SDI. It is noteworthy that the recently released GBD 2021 has updated its etiologies and methodologies [23]. Therefore, using the latest data is crucial for accurately interpreting the burden of HF in Asia.

From 1990 to 2021, the cases of HF in Asia increased significantly. The study results show that the crude prevalence and crude YLD rate of HF in Asia have exhibited a continuous upward trend, while the age-standardized rates have remained relatively stable. This difference clearly indicates that population growth and aging are the main driving factors behind the increase in crude rates. GBD 2021 data revealed that the proportion of people aged 65 and above in Asia rose from 4.83% in 1990 to 9.60% in 2021 [24]. The trend of population aging is unlikely to reverse in the short term, therefore it is necessary to prepare for the continued increase in the number of HF patients. Unfortunately, over the past three decades, most Asian countries have experienced an increase in age-standardized prevalence and YLD rates of HF, underscoring the severity of the HF burden in Asia. In 2021, approximately 29.54 million individuals lived with HF and this illness accounted for 2.85 million YLDs in Asia. Undoubtedly, older adults bear the heaviest burden of HF. In this population, HF is more likely to impair appetite and gastrointestinal function, reduce muscle mass and physical activity, leading to malnutrition and frailty—both of which are associated with poor prognosis [25,26]. Thus, special attention should be paid to nutrition and physical conditions in the management of elderly HF patients. Additionally, the greater HF burden observed in males compared to females across most age groups is closely linked to males generally bearing a heavier burden of risk factors and causes of HF [27]. This study found that the primary causes of HF among patients in Asia include ischemic heart disease, hypertensive heart disease, chronic respiratory diseases, and chronic obstructive pulmonary disease. Notably, these conditions share common risk factors, such as smoking, alcohol consumption, hypertension, and hyperglycemia, which are significantly more prevalent among men than women in Asia [28]. This disparity may help explain the higher burden of HF observed in men compared to women. Specifically, risk factors such as smoking and alcohol consumption promote the development and progression of cardiovascular diseases through multiple pathophysiological mechanisms, including direct cardiotoxicity, accelerated atherosclerosis, and exacerbation of COPD progression [29]. Based on these findings, we recommend incorporating tobacco and alcohol control into primary prevention strategies for HF. Additionally, policymakers in various countries should implement targeted health interventions for men, focusing on stronger tobacco and alcohol control measures.

Overall, the current age-standardized prevalence rate of HF in high-middle SDI locations are higher than in other regions. Regions with higher SDI generally have better nutritional status and healthcare resources. However, on the other hand, changes in dietary patterns, unhealthy lifestyles, and aging populations also contribute to the disease burden [30–33]. This explains the greater variability in the burden of HF among high SDI countries. For instance, Kuwait has the highest age-standardized prevalence rate, while Japan, Cyprus, and Brunei have significantly lower age-standardized prevalence rate compared to other Asian countries. Furthermore, Japan and Cyprus are the only two Asian countries with their EAPC less than zero, which may be attributed to their long-standing healthy lifestyles, advanced healthcare systems, and effective public health policies. For countries with lower SDI, the lack of adequate healthcare resources is an important factor, implying limited prevention, early diagnosis, and treatment capabilities, as well as poor accessibility and affordability of cardiovascular disease (CVD) medications [34,35]. Additionally, the prevalence of HF in these countries may be underestimated, as some of the population, due to limited healthcare resources, may not receive a timely and correct diagnosis. In contrast, in developed countries with sufficient medical resources, individuals with poorer socioeconomic conditions are more likely to develop HF compared to wealthier individuals [36]. For these countries, it’s crucial to strengthen primary prevention, focusing on low-cost and high-impact interventions. This includes measures such as implementing national public policies for salt intake control, offering free community blood pressure screenings (using simple electronic blood pressure monitors), and providing low-cost, long-acting antihypertensive medications to reduce the risk of HF prevalence.

In terms of the changing patterns of HF burden, most Asian countries show an increasing trend. However, the study found a downward trend in the burden of HF in some middle and lower-middle SDI regions between 2019 and 2021. This phenomenon requires a cautious interpretation, considering the specific context of the COVID-19 pandemic. Pandemic control measures led to restricted routine medical services, a decrease in non-emergency medical visits, and interruptions in chronic disease follow-up. At the same time, the reallocation of healthcare resources toward pandemic response reduced the accessibility of specialized care and halted early screening programs. Furthermore, SARS-CoV-2 infection may have influenced the natural history of the disease through mechanisms such as inducing myocardial injury and increasing cardiac load [37,38]. The adjustment of routine disease surveillance networks and changes in cause-of-death classification standards may have also interfered with data accuracy [39,40]. It’s crucial to emphasize that this downward trend likely reflects the unique circumstances during the pandemic. As global health systems gradually return to normal, we recommend that future studies continue to monitor this trend to confirm its sustainability. Meanwhile, our research results indicate that the associations between prevalence, YLDs, SDI, and EAPC are not significant. This reflects the complexity and diversity of HF burden across different countries. More than half of the Asian countries have EAPCs for age-standardized prevalence rate higher than the global average. Among these, Central and South Asian countries generally exhibit higher EAPCs, while the Middle Eastern and East Asian regions show divergent trends. It’s recommended that these regions strengthen the combined prevention and control of hypertension and CVD. For rural and resource-poor areas, they should promote simple blood pressure monitoring devices and mobile medical screenings to reduce the rate of missed diagnoses. South Korea has the highest EAPC, followed by Bhutan, India, and Nepal from South Asia. The rapid increase in HF burden observed in South Asia aligns with findings from previous studies [41]. These regions should improve their HF registry systems and develop differentiated prevention and control strategies based on registry data. Notably, in some Middle Eastern countries such as Afghanistan, Yemen, and Syria, war and instability have further exacerbated the already fragile social and healthcare systems, contributing to a greater HF burden. Considering the inequalities between regions, it is essential to learn from the experiences of countries that have successfully controlled HF to reverse the rising disease burden in Asia. In countries with limited healthcare resources, the widespread use of expensive individual-level prevention and early diagnosis measures may be unaffordable. Policies aimed at reducing population exposure to preventable risk factors, such as restricting the use of tobacco, promoting early screening of cardiovascular risk factors, and encouraging healthy lifestyles and dietary patterns, may be the most cost-effective and rapid measures [9,41–43].

We have updated the changing patterns of HF in Asia using the latest GBD 2021 data,

revealing for the first time the trends in Asia’s HF burden following the global public health event (the 2019 COVID-19 pandemic). Additionally, our study includes 48 Asian countries, adding an analysis of smaller Central Asian countries (such as Tajikistan and Kyrgyzstan) that were not included in Feng et al.’s study [44]. We found that the HF burden in these countries was significantly underestimated. At the same time, the latest 2021 data for Asia shows significant changes in both trend turning points and the national HF disease burden compared to the 2019 data. Meanwhile, this study serves as a continuation and refinement of the work by Ran J et al [16]. While the study by Ran J et al. primarily focused on global and regional trends in the burden of HF, it provided only a general overview of the situation in Asia. Therefore, by updating the data to 2021, identifying trend inflection points, and providing more detailed regional and etiological stratification, our study supplements the recent developments and region-specific characteristics that were not covered by the studies of Feng et al. and Ran J et al. This provides more precise evidence to support the formulation of HF prevention and control strategies in Asia.

However, this study also has some inevitable limitations. Although the data processing and modeling methods in GBD 2021 have been improved, the lack of raw data from some countries has led to these data being estimated only by Bayesian meta-regression disease Modelling-Meta Regression (DisMod-MR) [23]. This approach may lead to biases in data sources, especially in areas with incomplete medical records, rural, or resource-limited regions. Additionally, the assumptions of the model may introduce systematic errors, resulting in some discrepancies between the estimates and the actual data. Next, as mentioned earlier, due to insufficient healthcare resources in certain regions and the specific context of the COVID-19 pandemic, the prevalence of HF in low-SDI regions may be underestimated, and some data may be biased. Third, this study did not use joinpoint regression and only calculated the overall EAPC to reflect long-term trends. A single EAPC reflects only the overall average trend and may mask short-term fluctuations or turning points. Lastly, in the GBD study, HF is categorized as an “impairment” rather than a underlying cause of death. Consequently, mortality (Years of Life Lost, YLLs) is not estimated for HF, and the Disability-Adjusted Life Years (DALYs) for HF are equivalent to the YLDs. This precluded our analysis of the mortality burden (YLLs) attributable to HF, limiting our assessment to the non-fatal burden (prevalence and YLDs).

Our findings provide insights regarding the burden of HF over the past 30 years in Asian regions and countries with different SDI, which may help policy makers and clinicians to develop appropriate prevention and management strategies. HF is a challenging public health issue in both Asia and globally, with a continuously increasing burden. The HF burden in Asia is marked by complexity and diversity, as well as geographic and SDI-related inequalities. In underdeveloped regions with heavy HF burden, it is essential to promote accessible and cost-effective prevention and monitoring measures.

## Supporting information

S1 Fig Distribution of the EAPC in age-standardized prevalence (A) and YLD rate (B) in different SDI regions across 48 Asian countries, 1990–2021. (PDF)

S2 Fig Distribution of the correlation between prevalence (A), YLDs (B), SDI, and EAPC in 48 Asian countries, 2021. (PDF)

S1 Table The prevalence rate of four types of HF in males and females across different age groups. (DOCX)

S2 Table The prevalence number of four types of HF in males and females across different age groups. (DOCX)

S3 Table Attributable numbers and proportions of HF Cases in Asia, 2021. (DOCX)
