# Supplementary figures and images for: Burden of heart failure in Asian Countries from 1990 to 2021: Update from the Global Burden of Disease Study 2021
Source: PLoS One. 2026 Jul 29;21(7):e0352930. doi: 10.1371/journal.pone.0352930 (PMC13419183; doi:10.1371/journal.pone.0352930)

A

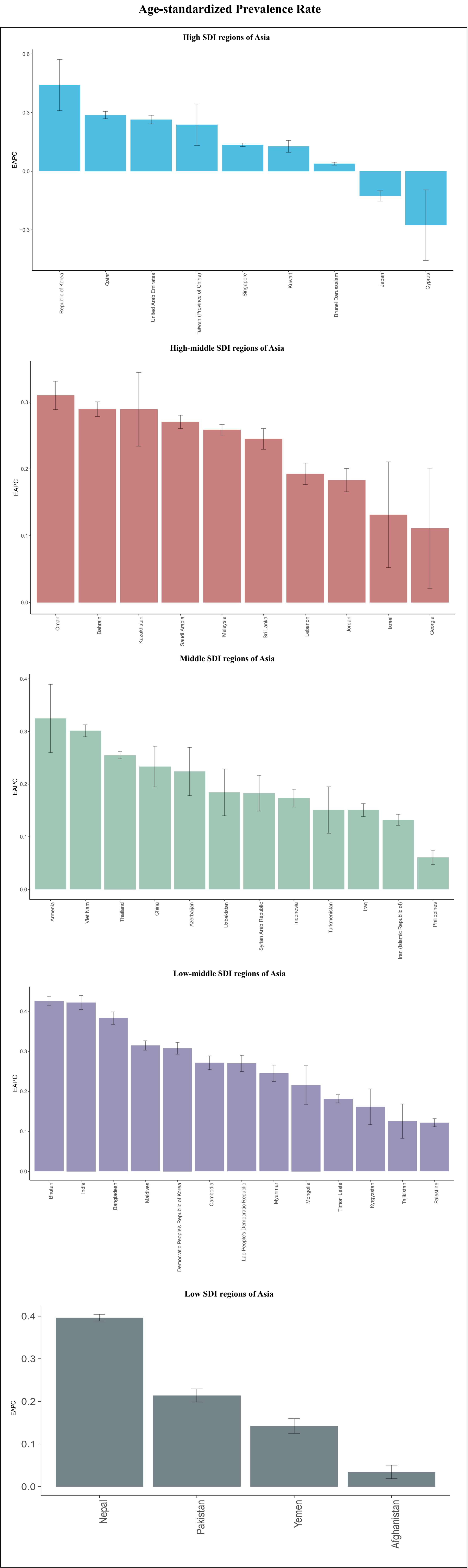

B

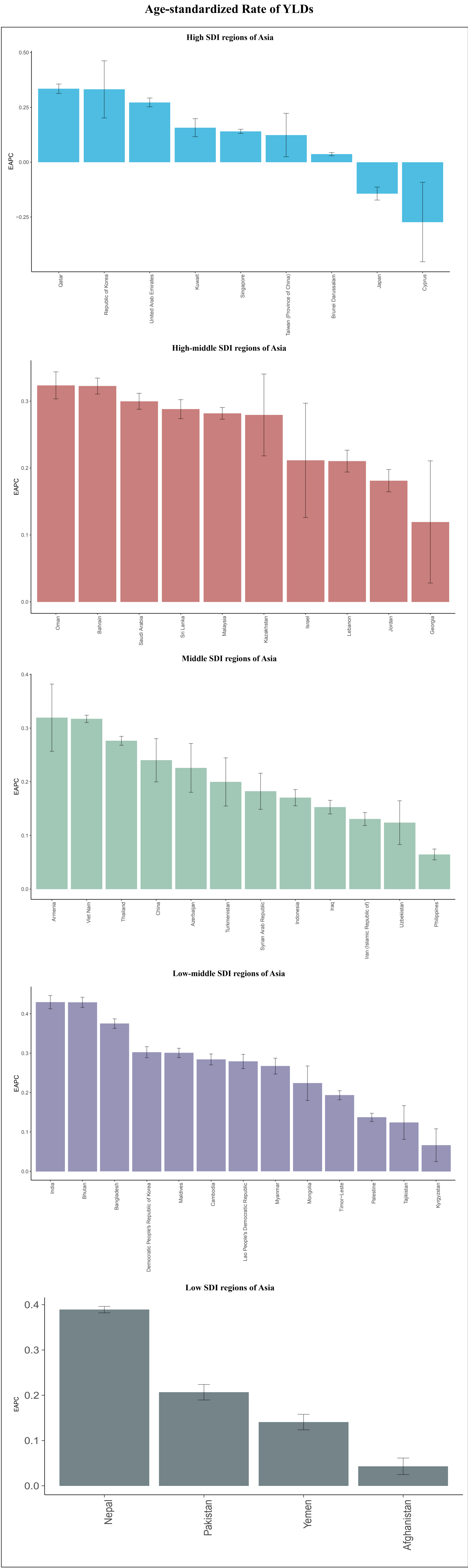

Supplement: S1 Fig — (PDF) [file pone.0352930.s001.pdf]

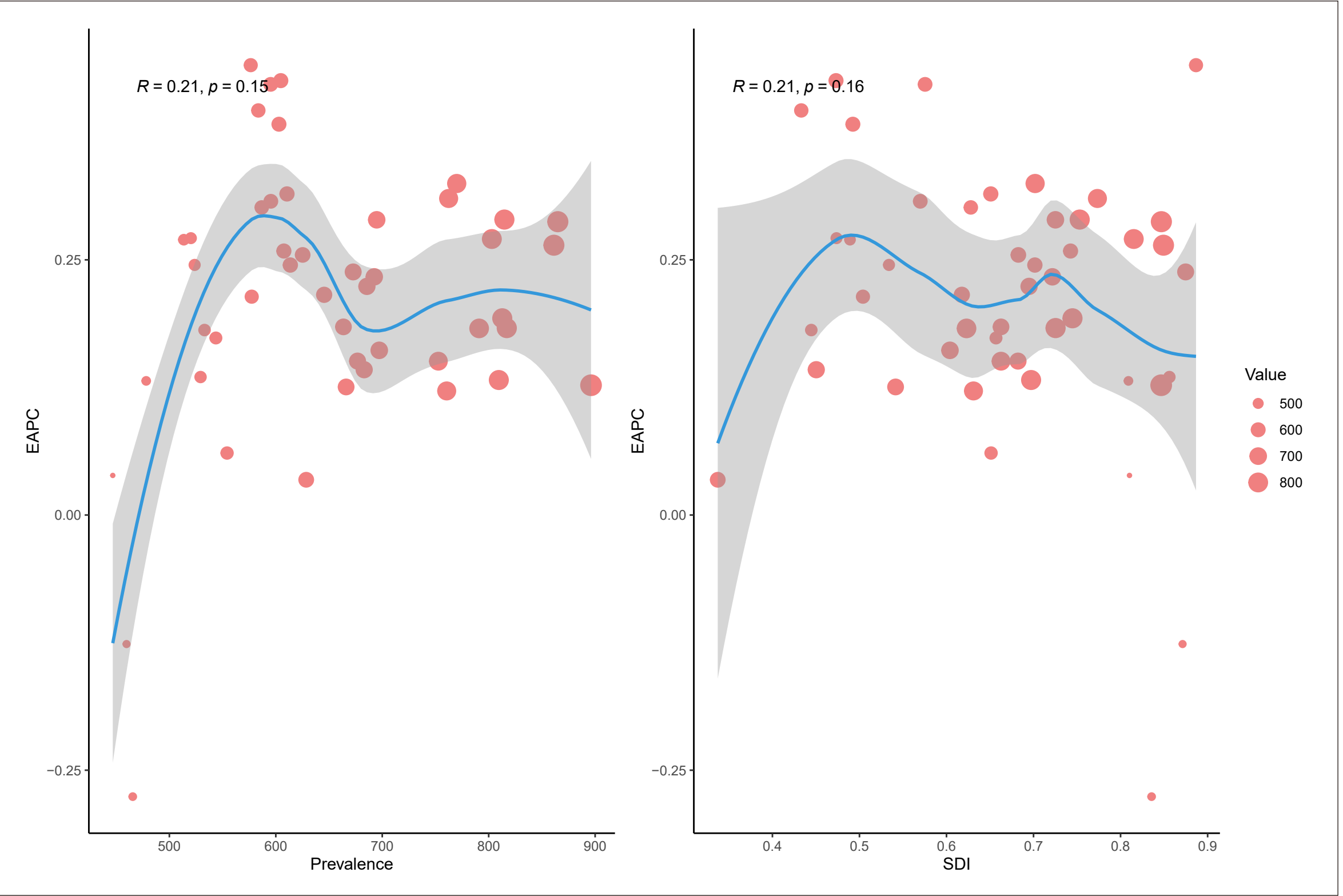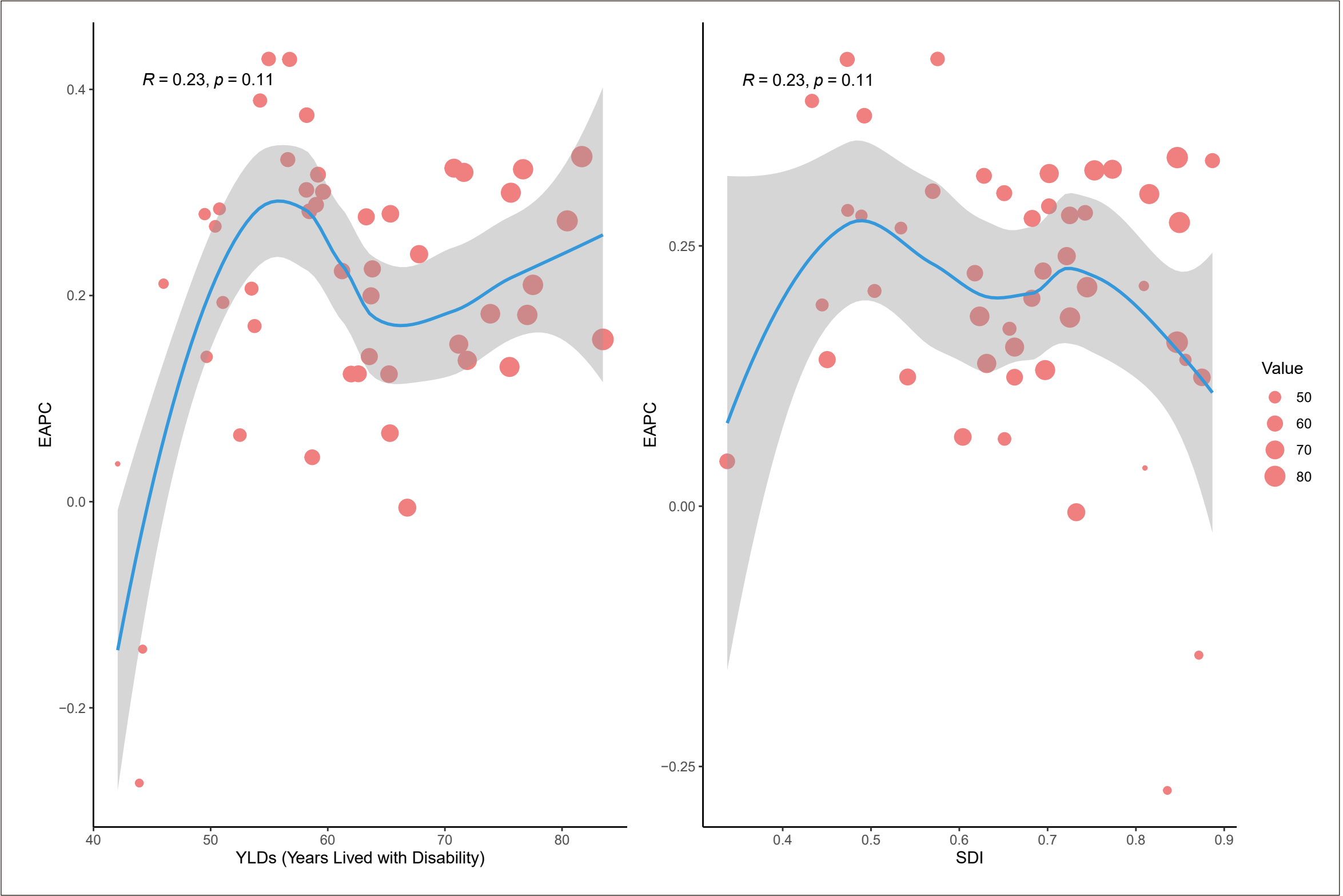

Supplement: S2 Fig — (PDF) [file pone.0352930.s002.pdf]
