# Supplementary material for: Burden of heart failure in Asian Countries from 1990 to 2021: Update from the Global Burden of Disease Study 2021
Source: PLoS One. 2026 Jul 29;21(7):e0352930. doi: 10.1371/journal.pone.0352930 (PMC13419183; doi:10.1371/journal.pone.0352930)
Supplement: S1 Table — (DOCX) [file pone.0352930.s003.docx]

**S1 Table: The prevalence rate of four types of HF in males and females across different age groups.**

|  | **Treated heart failure** | | **Severe heart failure** | | **Moderate heart failure** | | **Mild heart failure** | | **Heart failure** | |
| --- | --- | --- | --- | --- | --- | --- | --- | --- | --- | --- |
|  | **Male** | **Female** | **Male** | **Female** | **Male** | **Female** | **Male** | **Female** | **Male** | **Female** |
| <5 years | 132.75(110.93-159.88) | 109.95(91.15-132.81) | 117.72(96.74-141.84) | 97.5(79.33-117.29) | 43.62(33.7-55.06) | 36.12(27.93-45.62) | 67.54(52.63-85) | 55.94(43.64-70.34) | 361.63(300.65-433.31) | 299.52(249.45-356.65) |
| 10-14 years | 56.18(44.06-70.68) | 49.43(38.78-61.91) | 49.81(38.68-62.72) | 43.84(33.82-55.68) | 18.44(13.32-24.61) | 16.23(11.71-21.87) | 28.58(21.26-38.66) | 25.15(18.68-33.91) | 153(120.34-193.73) | 134.65(106.65-169.31) |
| 15-19 years | 51.5(39.64-65.83) | 45.45(34.78-58.27) | 45.64(35.05-58.7) | 40.28(30.83-51.92) | 16.89(11.93-23.08) | 14.9(10.4-20.53) | 26.18(18.46-37.51) | 23.1(16.38-33.3) | 140.2(110.59-178.72) | 123.73(96.75-158.17) |
| 20-24 years | 49.91(38.76-62.51) | 44.09(33.8-55.42) | 44.23(34.44-55.49) | 39.08(30.19-48.89) | 16.36(11.56-22.36) | 14.46(10.29-19.85) | 25.36(17.87-35.52) | 22.4(15.86-31.45) | 135.86(107.56-169.36) | 120.03(94.89-148.84) |
| 25-29 years | 47.79(35.91-60.76) | 42.5(31.63-53.75) | 42.36(31.24-55.07) | 37.68(28.08-49.2) | 15.68(10.5-22.06) | 13.94(9.29-19.61) | 24.26(16.42-34.96) | 21.58(14.94-30.56) | 130.09(98.89-166.78) | 115.7(87.47-147.46) |
| 30-34 years | 48.02(33.09-65.49) | 42.62(29.38-58.32) | 42.58(29.64-59.51) | 37.79(26.47-52.69) | 15.77(9.9-23.49) | 13.99(8.74-20.83) | 24.37(15.84-36.46) | 21.63(13.94-32.37) | 130.73(93.49-177.14) | 116.04(81.62-158.95) |
| 35-39 years | 59.85(42.36-76.75) | 51.55(37.51-66.39) | 53.07(38.45-69.78) | 45.7(33.27-59.84) | 19.67(13.05-28.6) | 16.94(11.44-24.4) | 30.39(19.91-43.45) | 26.17(17.44-37.25) | 162.98(118.92-210.02) | 140.36(103.57-179.32) |
| 40-44 years | 85.25(57.6-114.66) | 69.76(47.44-93.27) | 75.57(51.4-101.52) | 61.84(42.55-83.18) | 28.02(17.53-42.09) | 22.94(14.4-33.94) | 43.28(27.27-63.75) | 35.43(22.17-52.58) | 232.12(162.15-313.65) | 189.96(131.31-256.12) |
| 45-49 years | 129.02(100.9-164.88) | 100.41(78.2-127.36) | 114.38(87.98-146.87) | 89.01(68.55-115.63) | 42.38(28.89-59.13) | 32.98(22.37-45.88) | 65.48(44.83-93.08) | 50.97(35.1-72.26) | 351.25(274.65-444.13) | 273.37(210.47-348.9) |
| 50-54 years | 167.26(128.91-223.32) | 128.8(98.74-172.25) | 148.3(112.08-198.32) | 114.2(86.55-152.32) | 54.9(36.25-77.24) | 42.28(28.07-59.4) | 84.89(57.43-125.36) | 65.38(45.16-97.66) | 455.35(355.71-598.55) | 350.65(271.17-463.72) |
| 55-59 years | 193.95(139.48-263.19) | 149.41(107.81-207.28) | 171.97(120.84-231.88) | 132.48(94.31-179.08) | 63.62(39.76-92.52) | 49.01(30.43-72.29) | 98.47(66.45-153.33) | 75.86(50.74-119.04) | 528.02(387.61-723.57) | 406.77(297.15-556.45) |
| 5-9 years | 90.87(65.94-120.17) | 77.43(56.29-102.77) | 80.58(57.83-106.53) | 68.67(49.52-90.62) | 29.87(20.59-41.77) | 25.45(17.55-35.64) | 46.22(30.26-65.04) | 39.39(25.83-55.28) | 247.54(179.76-330.75) | 210.93(152.74-282.22) |
| 60-64 years | 374.4(300.85-467.13) | 285.61(226.48-361.39) | 331.95(261.58-415.1) | 253.24(196.53-318.08) | 122.86(84.35-168.86) | 93.73(64.41-130.24) | 190.03(136.63-262.82) | 144.97(104.54-200.96) | 1019.24(823.35-1238.63) | 777.55(613.43-958.16) |
| 65-69 years | 959.22(761.75-1205.37) | 745.13(591.8-938.82) | 850.44(673.89-1055.53) | 660.62(516.87-826.1) | 314.83(221.69-434.22) | 244.58(171.33-338) | 486.63(344.64-669.42) | 378.02(266.63-516.31) | 2611.11(2113.5-3223.69) | 2028.34(1640.66-2506.67) |
| 70-74 years | 1550.58(1185.54-1970.15) | 1227.24(940.19-1554.85) | 1374.7(1064.57-1748.63) | 1088.02(842.98-1387.14) | 508.91(353.36-706.24) | 402.79(278.79-559.35) | 786.3(540.01-1106.12) | 622.35(425.96-864.91) | 4220.49(3320.03-5301.27) | 3340.4(2602.73-4177.9) |
| 75-79 years | 2105.52(1577.05-2735.58) | 1684.29(1261.3-2181.76) | 1866.47(1414.49-2432.75) | 1493.06(1130.26-1941.48) | 691.11(477.51-983.94) | 552.82(379.12-781.22) | 1067.33(724.14-1497) | 853.79(574.3-1206.76) | 5730.43(4416.37-7342.71) | 4583.96(3521.63-5902.55) |
| 80-84 years | 2827.95(2211.92-3577.66) | 2353.68(1840.36-2968.54) | 2506.7(1996.74-3201.54) | 2086.36(1656.55-2650.81) | 927.7(646.92-1289.34) | 772.03(541.07-1087.44) | 1433.17(1009.6-1989.34) | 1192.84(839.36-1659.06) | 7695.52(6326.41-9561.88) | 6404.91(5247.95-7933.58) |
| 85-89 years | 3853.56(3050.15-4874.19) | 3476.86(2723.35-4413.92) | 3416.19(2602.35-4295.65) | 3082.36(2351.75-3873.22) | 1262.88(883.77-1743.15) | 1139.4(793.56-1577.46) | 1953.16(1393.58-2798.81) | 1762.35(1245-2514.04) | 10485.79(8465.38-13003.08) | 9460.96(7577.08-11723.64) |
| 90-94 years | 4636.07(3493.52-6015.64) | 4332.74(3252.53-5641.31) | 4110.87(3021.47-5357.47) | 3841.95(2808.18-5004.61) | 1519.03(1049.9-2148.75) | 1419.67(976.61-2037.43) | 2350.68(1555.21-3400.17) | 2197.08(1455.82-3197.3) | 12616.66(9796.55-16331.76) | 11791.44(9073.51-15411.02) |
| 95+ years | 5250(3833.5-7174.34) | 4908.33(3586.43-6734.08) | 4655.95(3297.91-6334.41) | 4352.94(3072.77-5997.93) | 1720.28(1160.22-2558.88) | 1608.38(1091.77-2388.78) | 2662.98(1695.33-3959.94) | 2489.97(1588.8-3674.88) | 14289.22(10589.26-19424.85) | 13359.62(9811.63-18239.38) |
