# Supplementary material for: Burden of heart failure in Asian Countries from 1990 to 2021: Update from the Global Burden of Disease Study 2021
Source: PLoS One. 2026 Jul 29;21(7):e0352930. doi: 10.1371/journal.pone.0352930 (PMC13419183; doi:10.1371/journal.pone.0352930)
Supplement: S2 Table — (DOCX) [file pone.0352930.s004.docx]

**S2 Table: The prevalence number of four types of HF in males and females across different age groups.**

|  | **Treated heart failure** | | **Severe heart failure** | | **Moderate heart failure** | | **Mild heart failure** | | **Heart failure** | |
| --- | --- | --- | --- | --- | --- | --- | --- | --- | --- | --- |
|  | **Male** | **Female** | **Male** | **Female** | **Male** | **Female** | **Male** | **Female** | **Male** | **Female** |
| <5 years | 237475.46(198450.6,286021.66) | 179596.44(148899.73,216941.41) | 210599.35(173058.58,253737.67) | 159273.29(129584.34,191591.23) | 78031.33(60281.23,98492.84) | 59009.25(45629.25,74514.95) | 120825.4(94156.2,152067.12) | 91382.77(71279.81,114894.28) | 646931.53(537844.07,775164.5) | 489261.74(407481.51,582588.29) |
| 10-14 years | 108981.03(85469.44,137108.63) | 87790.25(68865.46,109953.11) | 96628.38(75033,121672.33) | 77846.43(60051.42,98876.44) | 35769.1(25831.65,47733.54) | 28816.69(20802.58,38843.29) | 55434.75(41251.54,74994.06) | 44663.16(33165.11,60215.19) | 296813.26(233457.52,375811.33) | 239116.53(189395.11,300677.33) |
| 15-19 years | 94723.03(72909.7,121079.18) | 76977.81(58918.26,98700.66) | 83953.5(64464.69,107970.93) | 68226.24(52216.97,87948.77) | 31058.25(21952.74,42447.33) | 25243.48(17609.76,34780.17) | 48153.14(33958.17,69000.98) | 39134.45(27746.73,56406.96) | 257887.92(203416.56,328733.88) | 209581.98(163883.23,267914.51) |
| 20-24 years | 88970.46(69096.74,111438.45) | 74013.43(56742.5,93031.89) | 78855.22(61402.34,98929.51) | 65600.02(50682.98,82066.15) | 29171.64(20605.94,39868.57) | 24271.25(17275.76,33313.85) | 45205.07(31866.07,63329.18) | 37608.56(26625.82,52790.01) | 242202.38(191760.26,301930.08) | 201493.27(159283.22,249852.66) |
| 25-29 years | 86125.83(64713.18,109510.73) | 72571.27(54007.26,91784.39) | 76353.22(56301.03,99257.63) | 64336.74(47954.29,84008.76) | 28255.4(18917.4,39757.18) | 23809.76(15858.05,33491.49) | 43729.61(29592.23,63006.89) | 36852.36(25507.78,52187.22) | 234464.06(178238.37,300586.7) | 197570.13(149361.34,251801.84) |
| 30-34 years | 92387.08(63662.53,126011.46) | 77705.06(53561.91,106324.97) | 81923.8(57036.92,114496.43) | 68906.69(48259.69,96066.89) | 30334.1(19044,45202.05) | 25511.97(15933.24,37978.17) | 46881.66(30478.66,70153.39) | 39439.88(25424.14,59013.61) | 251526.63(179872.69,340829.73) | 211563.59(148805.81,289797.47) |
| 35-39 years | 105624.99(74747.98,135443.32) | 86464.24(62911.97,111366.74) | 93651.44(67858.53,123145.29) | 76661.66(55811.33,100373.65) | 34714.08(23028.43,50463.07) | 28417.05(19197.46,40922.32) | 53624.25(35135.83,76674.24) | 43901.54(29247.67,62487.94) | 287614.75(209861.63,370631.71) | 235444.49(173739.07,300795.09) |
| 40-44 years | 132342.19(89418.02,178012.07) | 103442.73(70341.58,138299.61) | 117323.22(79790.39,157608.44) | 91697.71(63089.68,123332.12) | 43503.82(27208.53,65337.95) | 34008.06(21354.12,50330.96) | 67189.93(42334.31,98974.58) | 52529.26(32875.9,77961.2) | 360359.17(251734.7,486929.41) | 281677.75(194700.44,379778.51) |
| 45-49 years | 195417.82(152827.05,249742.17) | 146968.4(114459.65,186425.2) | 173255.48(133264.4,222463.86) | 130291.47(100336.74,169254.07) | 64185.3(43753.57,89568.23) | 48278.84(32741.75,67153.54) | 99176.72(67907.67,140989.15) | 74603.33(51383.12,105761.34) | 532035.32(416007.21,672715.8) | 400142.04(308062.28,510695.8) |
| 50-54 years | 239778.73(184802.15,320144.25) | 181262.02(138964.05,242411.82) | 212602.62(160677.81,284304.37) | 160713.15(121806.88,214364.72) | 78698.73(51974.26,110723.93) | 59495.6(39500.11,83600.78) | 121698.2(82330.53,179718.01) | 92011.73(63556.86,137440.06) | 652778.27(509944.66,858072.89) | 493482.51(381618.62,652603.05) |
| 55-59 years | 238208.98(171315.18,323251.08) | 184843.11(133377.1,256429.18) | 211218.09(148414.08,284801.11) | 163900.29(116677.29,221542.91) | 78143.29(48830.33,113630.02) | 60637.64(37644.99,89430.54) | 120942.54(81614.18,188321.56) | 93854.08(62769.49,147271.84) | 648512.9(476058.88,888693.24) | 503235.12(367614.95,688409.48) |
| 5-9 years | 178332.59(129416.41,235844.64) | 139268.02(101244.8,184852.94) | 158152.06(113501.36,209075.45) | 123512.35(89075.93,162994.48) | 58621.62(40409.68,81973.83) | 45776.98(31568.77,64100.76) | 90710.67(59390.08,127642.59) | 70846.02(46457.05,99437.33) | 485816.94(352787.7,649108.5) | 379403.37(274732.12,507637.81) |
| 60-64 years | 347713.04(279408.58,433842.99) | 270893.77(214807.93,342766.25) | 308295.09(242935.48,385519.68) | 240187.19(186398.01,301692.47) | 114101.54(78339.29,156824.71) | 88896.24(61093.58,123524.69) | 176489.31(126894.57,244090.16) | 137502.01(99151.06,190602.97) | 946598.99(764673.55,1150352.98) | 737479.2(581815.95,908782.44) |
| 65-69 years | 781705.73(620778.42,982302.25) | 637699.88(506481.4,803469.68) | 693058.24(549179.9,860196.8) | 565375.94(442349.46,706996.67) | 256564.11(180664.82,353862.27) | 209316.66(146628.2,289272.13) | 396572.98(280858.4,545539.3) | 323516.1(228190.25,441868.74) | 2127901.06(1722375.97,2627110.84) | 1735908.58(1404122.01,2145276.2) |
| 70-74 years | 895011.79(684305.25,1137194.86) | 766450.5(587181.25,971053.58) | 793490.76(614479.15,1009330.98) | 679505.17(526467.58,866316.5) | 293746.27(203961.37,407650.64) | 251556.73(174111.44,349330.5) | 453862.91(311698.85,638464.28) | 388675.2(266024.68,540161.88) | 2436111.72(1916359.15,3059952.95) | 2086187.59(1625489.31,2609238.5) |
| 75-79 years | 743934.71(557214.47,966552.05) | 683036.5(511500.5,884777.67) | 659473.4(499777.15,859556.22) | 605486.32(458360.97,787337.49) | 244188.37(168716.18,347653) | 224188.55(153746.45,316810.23) | 377116.86(255857.52,528930.72) | 346242.86(232900.23,489384.38) | 2024713.34(1560419.76,2594372.26) | 1858954.23(1428140.98,2393687.65) |
| 80-84 years | 572631.23(447892.22,724441.34) | 623469.99(487494.83,786341.14) | 507582.97(404320.22,648279.82) | 552659.99(438806.39,702178.49) | 187850.48(130993.96,261078.3) | 204505.46(143324.41,288053.92) | 290201.94(204434.8,402821.66) | 315973.36(222338.63,439472.28) | 1558266.61(1281035.51,1936185.88) | 1696608.8(1390138.6,2101540.1) |
| 85-89 years | 342696.81(271249.16,433461.32) | 498154.09(390193.48,632412.91) | 303801.4(231426.67,382011.6) | 441630.9(336952.01,554942.94) | 112307.7(78593.57,155018.23) | 163249.59(113698.52,226013.62) | 173694.39(123931.27,248898.3) | 252504.51(178380.09,360203.91) | 932500.3(752825.93,1156362.86) | 1355539.08(1085621.91,1679729.46) |
| 90-94 years | 124136.6(93543.44,161076.26) | 239438.43(179742.97,311753.16) | 110073.7(80903.63,143452.96) | 212316.1(155187.14,276567.65) | 40673.8(28112.41,57535.48) | 78454.55(53970.05,112593.87) | 62942.43(41642.63,91043.72) | 121416.2(80452.09,176691.13) | 337826.53(262314.62,437303.16) | 651625.28(501425.48,851652.45) |
| 95+ years | 33910.2(24760.93,46339.73) | 84188.39(61515.09,115504.1) | 30073.2(21301.47,40914.49) | 74662.33(52704.58,102877.43) | 11111.47(7493.93,16528.04) | 27587.26(18726.26,40972.66) | 17200.44(10950.3,25577.6) | 42708.42(27251.31,63032.08) | 92295.31(68396.96,125466.81) | 229146.4(168290.61,312844.78) |
