# Supplementary material for: Burden of heart failure in Asian Countries from 1990 to 2021: Update from the Global Burden of Disease Study 2021
Source: PLoS One. 2026 Jul 29;21(7):e0352930. doi: 10.1371/journal.pone.0352930 (PMC13419183; doi:10.1371/journal.pone.0352930)
Supplement: S3 Table — (DOCX) [file pone.0352930.s005.docx]

**S3 Table: Attributable numbers and proportions of HF Cases in Asia, 2021.**

| **Prevalence** | | |  | **YLDs** | | |
| --- | --- | --- | --- | --- | --- | --- |
| **attribution** | **number** | **percent** |  | **attribution** | **number** | **percent** |
| Cardiovascular diseases | 24088816.47 | 81.52827692 |  | Cardiovascular diseases | 2319047.09 | 81.20734528 |
| Ischemic heart disease | 10279354.56 | 34.79033793 |  | Ischemic heart disease | 917471.6695 | 32.12760921 |
| Hypertensive heart disease | 7266166.993 | 24.59224493 |  | Hypertensive heart disease | 647404.7143 | 22.67052635 |
| Chronic respiratory diseases | 2606721.396 | 8.822413672 |  | Stroke | 286529.1366 | 10.0335481 |
| Chronic obstructive pulmonary disease | 2556257.896 | 8.651620633 |  | Chronic respiratory diseases | 231348.1989 | 8.101246908 |
| Rheumatic heart disease | 1807834.061 | 6.118590182 |  | Chronic obstructive pulmonary disease | 226712.2464 | 7.938907214 |
| Cardiomyopathy and myocarditis | 1705728.235 | 5.77301438 |  | Rheumatic heart disease | 163476.906 | 5.724560578 |
| Congenital heart anomalies | 1684583.092 | 5.701448926 |  | Cardiomyopathy and myocarditis | 155356.0384 | 5.4401877 |
| Congenital birth defects | 1684583.092 | 5.701448926 |  | Congenital heart anomalies | 154702.4959 | 5.417302241 |
| Other cardiomyopathy | 1523183.042 | 5.155192619 |  | Congenital birth defects | 154702.4959 | 5.417302241 |
| Stroke | 1374053.618 | 4.650466079 |  | Intracerebral hemorrhage | 147111.2009 | 5.151473697 |
| Non-rheumatic valvular heart disease | 1030134.733 | 3.486477215 |  | Other cardiomyopathy | 138485.7989 | 4.849433258 |
| Chronic kidney disease | 801012.1906 | 2.711015038 |  | Ischemic stroke | 124558.9608 | 4.361749521 |
| Diabetes and kidney diseases | 801012.1906 | 2.711015038 |  | Chronic kidney disease | 101920.6689 | 3.569012025 |
| Non-rheumatic degenerative mitral valve disease | 702212.1718 | 2.376627696 |  | Diabetes and kidney diseases | 101920.6689 | 3.569012025 |
| Intracerebral hemorrhage | 699458.7838 | 2.367308892 |  | Non-rheumatic valvular heart disease | 91977.53781 | 3.220827944 |
| Ischemic stroke | 603875.9859 | 2.043810191 |  | Non-rheumatic degenerative mitral valve disease | 62547.54628 | 2.190261771 |
| Chronic kidney disease due to other and unspecified causes | 423129.7824 | 1.432077085 |  | Chronic kidney disease due to other and unspecified causes | 53641.01236 | 1.878376783 |
| Non-rheumatic calcific aortic valve disease | 323384.0897 | 1.094489123 |  | Cirrhosis and other chronic liver diseases | 36958.66474 | 1.294201856 |
| Atrial fibrillation and flutter | 293226.0607 | 0.992419678 |  | Digestive diseases | 36958.66474 | 1.294201856 |
| Digestive diseases | 238648.6037 | 0.807703005 |  | Non-rheumatic calcific aortic valve disease | 29009.77279 | 1.015851142 |
| Cirrhosis and other chronic liver diseases | 238648.6037 | 0.807703005 |  | Atrial fibrillation and flutter | 26163.86973 | 0.916194592 |
| Chronic kidney disease due to diabetes mellitus type 2 | 170302.2849 | 0.576385804 |  | Chronic kidney disease due to diabetes mellitus type 2 | 21663.30091 | 0.758595703 |
| Chronic kidney disease due to hypertension | 126462.4326 | 0.42801041 |  | Chronic kidney disease due to hypertension | 16140.52714 | 0.565201701 |
| Other cardiovascular and circulatory diseases | 118236.4718 | 0.400169756 |  | Subarachnoid hemorrhage | 14858.9749 | 0.520324883 |
| Endocarditis | 110699.9478 | 0.374662492 |  | Chronic hepatitis B including cirrhosis | 12335.12776 | 0.431945942 |
| Myocarditis | 106908.2563 | 0.361829562 |  | Cirrhosis due to other causes | 11035.2562 | 0.386427625 |
| Pulmonary Arterial Hypertension | 103381.7859 | 0.349894271 |  | Other cardiovascular and circulatory diseases | 10939.63135 | 0.383079078 |
| Hemoglobinopathies and hemolytic anemias | 86863.83048 | 0.293989472 |  | Endocarditis | 10152.4801 | 0.35551497 |
| Chronic hepatitis B including cirrhosis | 79712.15877 | 0.269784734 |  | Myocarditis | 9891.777866 | 0.346385817 |
| Alcoholic cardiomyopathy | 75636.93656 | 0.255992199 |  | Chronic kidney disease due to glomerulonephritis | 9659.666681 | 0.338257852 |
| Chronic kidney disease due to glomerulonephritis | 74806.68732 | 0.253182232 |  | Pulmonary Arterial Hypertension | 9575.106164 | 0.33529675 |
| Cirrhosis due to other causes | 71111.47978 | 0.240675851 |  | Hemoglobinopathies and hemolytic anemias | 8049.345955 | 0.281868367 |
| Subarachnoid hemorrhage | 70718.84857 | 0.239346996 |  | Alcoholic cardiomyopathy | 6978.461698 | 0.244368625 |
| Chronic hepatitis C including cirrhosis | 43066.89008 | 0.145759313 |  | Chronic hepatitis C including cirrhosis | 6663.489515 | 0.233339071 |
| Interstitial lung disease and pulmonary sarcoidosis | 41741.43121 | 0.141273315 |  | Cirrhosis due to alcohol | 5545.023316 | 0.194173126 |
| Thalassemias | 41456.08508 | 0.140307565 |  | Thalassemias | 3841.192764 | 0.134509156 |
| Cirrhosis due to alcohol | 35856.65782 | 0.121356378 |  | Interstitial lung disease and pulmonary sarcoidosis | 3826.39875 | 0.133991106 |
| Other hemoglobinopathies and hemolytic anemias | 35072.47144 | 0.11870231 |  | Other hemoglobinopathies and hemolytic anemias | 3250.246173 | 0.113815655 |
| Endocrine, metabolic, blood, and immune disorders | 27939.81265 | 0.094561922 |  | Endocrine, metabolic, blood, and immune disorders | 2587.709946 | 0.09061526 |
| Substance use disorders | 11546.35015 | 0.039078468 |  | Nonalcoholic fatty liver disease including cirrhosis | 1379.767945 | 0.048316092 |
| Drug use disorders | 11546.35015 | 0.039078468 |  | Substance use disorders | 1071.07693 | 0.037506489 |
| G6PD deficiency | 10335.27397 | 0.034979596 |  | Drug use disorders | 1071.07693 | 0.037506489 |
| Nonalcoholic fatty liver disease including cirrhosis | 8901.417198 | 0.030126727 |  | G6PD deficiency | 957.9070183 | 0.033543556 |
| Pneumoconiosis | 8722.068346 | 0.029519724 |  | Chronic kidney disease due to diabetes mellitus type 1 | 816.1618113 | 0.028579986 |
| Amphetamine use disorders | 7611.507652 | 0.025761046 |  | Pneumoconiosis | 809.5537599 | 0.028348589 |
| Chronic kidney disease due to diabetes mellitus type 1 | 6311.003475 | 0.021359507 |  | Amphetamine use disorders | 706.1576016 | 0.024727908 |
| Silicosis | 5697.85682 | 0.01928432 |  | Silicosis | 528.9480599 | 0.018522465 |
| Other non-rheumatic valve diseases | 4538.471329 | 0.015360395 |  | Other non-rheumatic valve diseases | 420.2187459 | 0.014715031 |
| Cocaine use disorders | 3934.842493 | 0.013317422 |  | Cocaine use disorders | 364.9193286 | 0.01277858 |
| Coal workers pneumoconiosis | 1387.66515 | 0.004696534 |  | Coal workers pneumoconiosis | 128.7754859 | 0.004509402 |
| Other pneumoconiosis | 859.8047311 | 0.002909998 |  | Other pneumoconiosis | 79.79378286 | 0.002794183 |
| Asbestosis | 776.7416453 | 0.002628872 |  | Asbestosis | 72.03643126 | 0.002522539 |
| Neglected tropical diseases and malaria | 446.9163281 | 0.001512582 |  | Communicable, maternal, neonatal, and nutritional diseases | 25.74646719 | 0.000901578 |
| Communicable, maternal, neonatal, and nutritional diseases | 446.9163281 | 0.001512582 |  | Neglected tropical diseases and malaria | 25.74646719 | 0.000901578 |
| Chagas disease | 446.9163281 | 0.001512582 |  | Chagas disease | 25.74646719 | 0.000901578 |
